# Gene set correlation enrichment analysis for interpreting and annotating gene expression profiles

**DOI:** 10.1093/nar/gkad1187

**Published:** 2023-12-14

**Authors:** Lan-Yun Chang, Meng-Zhan Lee, Yujia Wu, Wen-Kai Lee, Chia-Liang Ma, Jun-Mao Chang, Ciao-Wen Chen, Tzu-Chun Huang, Chia-Hwa Lee, Jih-Chin Lee, Yu-Yao Tseng, Chun-Yu Lin

**Affiliations:** Institute of Bioinformatics and Systems Biology, National Yang Ming Chiao Tung University, Hsinchu 300, Taiwan; Institute of Bioinformatics and Systems Biology, National Yang Ming Chiao Tung University, Hsinchu 300, Taiwan; Institute of Bioinformatics and Systems Biology, National Yang Ming Chiao Tung University, Hsinchu 300, Taiwan; Institute of Bioinformatics and Systems Biology, National Yang Ming Chiao Tung University, Hsinchu 300, Taiwan; Institute of Bioinformatics and Systems Biology, National Yang Ming Chiao Tung University, Hsinchu 300, Taiwan; Institute of Bioinformatics and Systems Biology, National Yang Ming Chiao Tung University, Hsinchu 300, Taiwan; Institute of Bioinformatics and Systems Biology, National Yang Ming Chiao Tung University, Hsinchu 300, Taiwan; Institute of Bioinformatics and Systems Biology, National Yang Ming Chiao Tung University, Hsinchu 300, Taiwan; School of Medical Laboratory Science and Biotechnology, College of Medical Science and Technology, Taipei Medical University, New Taipei City 235, Taiwan; Center for Intelligent Drug Systems and Smart Bio-devices (IDS^2^B), National Yang Ming Chiao Tung University, Hsinchu 300, Taiwan; TMU Research Center of Cancer Translational Medicine, Taipei Medical University, Taipei 110, Taiwan; Ph.D. Program in Medical Biotechnology, College of Medical Science and Technology, Taipei Medical University, New Taipei City 235, Taiwan; Department of Otolaryngology-Head and Neck Surgery, Tri-Service General Hospital, National Defense Medical Center, Taipei 110, Taiwan; Department of Food Science, Nutrition, and Nutraceutical Biotechnology, Shih Chien University, Taipei 104, Taiwan; Institute of Bioinformatics and Systems Biology, National Yang Ming Chiao Tung University, Hsinchu 300, Taiwan; Center for Intelligent Drug Systems and Smart Bio-devices (IDS^2^B), National Yang Ming Chiao Tung University, Hsinchu 300, Taiwan; Department of Biological Science and Technology, National Yang Ming Chiao Tung University, Hsinchu 300, Taiwan; Cancer and Immunology Research Center, National Yang Ming Chiao Tung University, Taipei 112, Taiwan; Institute of Data Science and Engineering, National Yang Ming Chiao Tung University, Hsinchu 300, Taiwan; School of Dentistry, Kaohsiung Medical University, Kaohsiung 807, Taiwan

## Abstract

Pathway analysis, including nontopology-based (non-TB) and topology-based (TB) methods, is widely used to interpret the biological phenomena underlying differences in expression data between two phenotypes. By considering dependencies and interactions between genes, TB methods usually perform better than non-TB methods in identifying pathways that include closely relevant or directly causative genes for a given phenotype. However, most TB methods may be limited by incomplete pathway data used as the reference network or by difficulties in selecting appropriate reference networks for different research topics. Here, we propose a gene set correlation enrichment analysis method, Gscore, based on an expression dataset-derived coexpression network to examine whether a differentially expressed gene (DEG) list (or each of its DEGs) is associated with a known gene set. Gscore is better able to identify target pathways in 89 human disease expression datasets than eight other state-of-the-art methods and offers insight into how disease-wide and pathway-wide associations reflect clinical outcomes. When applied to RNA-seq data from COVID-19-related cells and patient samples, Gscore provided a means for studying how DEGs are implicated in COVID-19-related pathways. In summary, Gscore offers a powerful analytical approach for annotating individual DEGs, DEG lists, and genome-wide expression profiles based on existing biological knowledge.

## Graphical Abstract

**Graphical Abstract:**
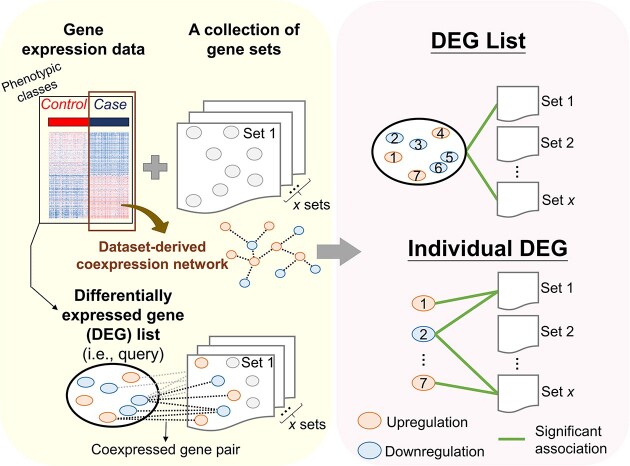

## Introduction

Recent advances in genome-/proteome-wide profiling technologies for DNA, RNA, and protein have lowered the costs and improve the accuracy of sequencing, which in turn has increased the amount of available data. Accordingly, the most common need is to interpret and annotate these profiles with the context of prior knowledge in biology, biomedicine, and pharmacology. Pathway analysis has emerged as the primary approach for capturing insight into the biological mechanisms associated with differentially expressed genes (DEGs) and proteins (1). Over 70 pathway analysis methods have been proposed; these can be categorized into nontopology-based (non-TB, also called gene set analysis methods) or topology-based (TB) methods (2,3). The first-generation non-TB methods are known as overrepresentation analysis (ORA), which assess whether the DEGs in a given list are over- or underrepresented in gene sets (e.g. groups of genes in the pathways) using Fisher's exact test, hypergeometric test, or *χ^2^* test (1,2,4). The early tools developed based on ORA were Onto-Express (5,6) and GeneMAPP (7), followed by EASE (8), GeneMerge (9), FuncAssociate (10), and so on. Some commonly used Gene Ontology (GO) analysis tools, such as FatiGO (11), GOstats (12), DAVID (13,14), and WebGestalt (15), are also based on ORA. However, the ORA methods often disregard the measured changes in gene expression (i.e. treating each gene equally) and exclude marginally or less significant genes, resulting in information loss (1,2). To overcome these limitations, functional class scoring (FCS) approaches, assuming that weak but coordinated changes in sets of functionally related genes (e.g. pathways) may also play an important role, have been developed as the second-generation of non-TB methods (1,2). Widely used FCS approaches include GSEA (16), GSA (17), PADOG (18), and so on. Although FCS approaches address some of the limitations of ORA, they are similar to ORA in that they analyze each pathway independently and fail to account for cross-talk and overlap between pathways, limiting their ability to describe and capture biological processes (1,2).

Thus, third-generation approaches, known as topology-based (TB, also called pathway topology-based) methods, have been proposed in order to utilize pathway/network knowledge bases that provide additional information, including the dependencies and interactions between genes (or proteins) (1,19,20). The earliest TB tools and methods were Pathway-Express (21,22) and SPIA (23), followed by more than 30 others, including CePaORA/CePaGSA (24,25), PathNet (26), ROntoTools (27,28), and NEA/NEArender/EviNet (29–31). Previous studies indicated that TB methods perform better than non-TB methods in identifying pathways that include genes that are closely relevant to or directly induce the phenotype, especially when researchers would like to consider how various genes (or proteins) interact, explain downstream effects, and further investigate fundamental mechanisms at the pathway level (1,2). Although the availability of pathway data has increased substantially, the current pathways from an individual database used as the reference network (i.e. global network) for some TB methods are still incomplete (1). Inconsistent pathway data formats among the different databases also have posed a challenge in pathway integration (1,19). Thus, the NEA method and related tools (29–31) offer a solution that allows users to choose and input a reference network. However, selecting appropriate reference networks for different research topics can be a challenging issue for users. Coexpression network analysis is an alternative solution that can be used to infer correlations between genes in a specific spatiotemporal state from genome-wide expression profiles (32,33). It can be effectively utilized to distinguish the correlations for examining which genes are active in the same biological processes simultaneously (34) and to further associate a group of genes of unknown function with previously annotated gene sets. Another unmet need is to prioritize DEGs based on their impacts on a specific pathway after associating a long list of DEGs with this pathway. The prioritization of DEGs can guide the planning of follow-up experiments and translation to clinical applications (35,36). Nevertheless, most of the previously proposed methods and tools were designed without consideration of the use of coexpression networks or the need for DEG prioritization.

To address these issues, inspired by the previous TB methods (e.g. Pathway-Express (21,22) and NEA (29)) and coexpression network analysis (32,34,35), we developed a gene set correlation enrichment analysis (Gscore) method that utilizes a coexpression network derived from the given gene expression data instead of a preexisting aggregate network (or pathway sets) as the reference. Based on the coexpression network, Gscore estimates the enrichment of coexpressed gene pairs between DEGs in a collection of gene sets and in a selected list (as well as each of its DEGs) using the hypergeometric distribution with Benjamini–Hochberg correction. Our experimental results demonstrated that Gscore provided a better ability to identify target pathways (i.e. the lowest median rank values) than the other five TB (i.e. CePaORA, CePaGSA, NEA, ROntoTools, and SPIA) and three non-TB (i.e. ORA, GSEA, and GSA) methods on 75 microarray datasets related to 15 diseases and 16 RNA sequencing (RNA-seq) datasets in 16 cancer types. In the analyses of DEGs derived from RNA-seq datasets for 16 cancers, Gscore displayed the most efficient ability to associate the DEGs with pancancer pathways, which are common hallmarks of cancer, defined by the Kyoto Encyclopedia of Genes and Genomes (KEGG) database (37). Moreover, Gscore also achieved high accuracy, precision, and a low false positive rate (FPR) in detecting associations between individual DEGs and pancancer pathways. The use of Gscore in conjunction with overall survival data and a meta-analytical framework facilitates cancer-wide and pathway-wide investigations to quantify common signatures and prognostic correlations in individual DEGs associated with pancancer pathways. By applying pathway analysis methods to four RNA-seq datasets of samples derived from patients with coronavirus disease 2019 (COVID-19) or cells infected with SARS-CoV-2, we observed that Gscore not only facilitates better associations with the target and related pathways of COVID-19 but also offers insights for studying how individual DEGs are implicated in those pathways. Here, Gscore was implemented through a user-friendly, freely available online tool to provide statistically rigorous hypothesis testing, a clear illustration of the Gscore procedure, and intuitive and interactive visualizations of findings. In summary, Gscore offers a new and valuable framework to facilitate the annotation and characterization of individual DEGs, DEG lists, and genome-wide expression profiles based on prior biological knowledge.

## Materials and methods

### Gene expression datasets

To examine the performance of different pathway analysis methods, we first assembled 75 microarray expression datasets in 15 diseases from the Gene Expression Omnibus (GEO) database (38) (Supplementary Table S1), which were used as benchmark datasets to evaluate different methods in previous studies (2,18). To independently test the performance of these methods on RNA-seq data, RNA-seq datasets in 16 cancer types were further collected from The Cancer Genome Atlas (TCGA) database (39) (Supplementary Table S2).

For microarray data, the SOFT format file, the corresponding annotation file retrieved from the GEO, and the *Bioconductor AnnotationData* package (v. 3.14) were used to retrieve information about the array platform, including the probe ID, Entrez gene ID, and gene description. We downloaded raw CEL files of Affymetrix data and employed the *threestep* function from the *affyPLM* package (v. 1.70.0) for robust multiarray average (RMA) background adjustment, quantile normalization, and median polish summarization. The median value was used if more than one probe was mapped to the same gene. For the TCGA datasets, we downloaded level 3 RNASeqV2 data (upper quartile-normalized RNA-seq by expectation maximization (RSEM) count estimates) containing the expression profiles of 20531 genes with Entrez Gene IDs for 7351 samples, comprising 6649 tumor samples and 702 corresponding normal tissues. RNA-seq data were matched through the patient barcodes provided by TCGA. The values of both RNA-seq and microarray data were log_2_-transformed before being used for further analysis.

To identify DEGs between case and corresponding control samples for the microarray datasets in each disease, we followed the criteria suggested by previous work (2) to evaluate the *P* value for each gene based on the two-sample *t* test using the *ttest_ind* function from the *SciPy* package (v. 1.9.1). Among the genes with *P* values <0.05, the top 400 genes ranked by the absolute log_2_(fold change) values were considered DEGs (2). For the TCGA RNA-seq datasets, a modified t-statistic (*limma* package v. 3.24.15) was utilized to measure DEGs between tumors and corresponding normal samples in each cancer type. Based on the criteria suggested by previous studies (35,40), we used |log_2_(fold change)| ≥ 1 and adjusted *P* values ≤0.05 to identify DEGs.

Additionally, to further apply and validate our Gscore method and the other pathway analysis methods, we assembled four RNA-seq datasets relevant to coronavirus disease 2019 (COVID-19), including two patient-derived datasets (GEO ID: GSE157103 (41) and GSE150316 (42)) and two cell-derived datasets (GSE147507 (43,44) and GSE160435 (45); Supplementary Table S3). The patient-derived plasma and leukocyte (PPL) sample dataset (GSE157103) consists of 100 plasma and leukocyte samples from hospitalized patients with COVID-19 and 26 samples from patients without COVID-19. Another patient-derived autopsy (PA) sample dataset (GSE150316) comprises five control autopsy lung samples from five patients and 29 autopsy samples from eight patients with high SARS-CoV-2 loads. The normal human bronchial epithelial (NHBE) cell dataset (GSE147507) contains three mock-treated and three SARS-CoV-2-infected primary human lung epithelium samples. The alveolar type 2 progenitor (AT2) cell dataset (GSE160435) includes five mock-transfected and five SARS-CoV-2-infected organoids generated from primary lung AT2 cells. For the NHBE cell, AT2 cell, and PA sample datasets, we followed the DEG identification criteria from the corresponding works, i.e. *P* values < 0.05 (Wald test; *DESeq2*). For the PPL sample dataset, we defined the DEGs based on the same criteria used in the TCGA datasets. Note that either the gene expression data or the corresponding DEG list was used as input depending on the requirement of the pathway analysis method used; for instance, gene expression data were used for the PADOG, GSA, and GSEA methods. In the online Gscore tool, the DEG criteria can be set by the user.

### KEGG pathway set

To assess the pathway analysis methods based on their ability to identify the target pathway depicting the mechanism corresponding to the condition studied, we first collected 347 human pathways containing 8146 proteins from the KEGG database (v. 102) (37). A collected expression dataset is relevant to a disease or condition for which the mechanisms are known and are described in a KEGG pathway, called the target pathway (Supplementary Table S4 and S5). The ability to rank the target pathway on top based on its statistical significance (e.g. *P* value or FDR *q* value) was used to evaluate the pathway analysis methods (2,3,18,46). For each KEGG pathway, the corresponding gene set includes all the genes (without interactions) involved in the pathway; in other words, the gene set is extracted from the original pathway without the structure or other additional information (Supplementary Figures S1A and S1B). Note that the pathway or corresponding gene set was used as input depending on the requirement of the pathway analysis method used.

KEGG pathways consist of manually curated pathway maps encompassing experts' understanding of molecular interactions, reactions, and relation networks for numerous categories (37). To evaluate whether various methods could detect the associations between pancancer pathways and the DEG lists (or their individual DEGs) derived from the TCGA RNA-seq datasets related to 16 cancers, we first collected all 11 pathways that belong to the category ‘6.1 Cancer: overview’. Then, we obtained the related pathways, as defined in the KEGG database (Supplementary Table S6). For example, ‘PD-L1 expression and PD-1 checkpoint pathway in cancer (hsa05235)’ is one of the pathways in the category of ‘6.1 Cancer: overview’, and ‘MAPK signaling pathway (hsa04010)’ is defined as a related pathway, recorded at the bottom of the *pathway entry* web page (https://www.genome.jp/entry/hsa05235, Supplementary Figure S1C). Finally, these 11 pathways and 58 nonredundant related pathways (69 in total) were deemed pancancer pathways. Using a parallel procedure, we also collected the ‘coronavirus disease - COVID-19 (hsa05171)’ pathway as the target pathway and identified its 16 related pathways. These 17 COVID-19-related pathways (Supplementary Table S7) were used to examine the ability to detect associations between these pathways and the DEG lists (or their individual DEGs) derived from four RNA-seq datasets relevant to COVID-19.

### Prognostic genes in 16 cancers

For the prognostic gene sets in 16 cancers, we chose TCGA patient samples with both gene expression data and clinical outcome data to assess the correlation of each gene with survival outcomes. For each cancer, we estimated the correlation between each gene and the 10-year survival outcomes by Cox proportional hazards regression. The *coxph* function of the R survival package (v. 3.2-12) was used for Cox proportional hazards regression analysis. We utilized the median (50%) expression value of each gene in every malignancy to stratify the corresponding patients into high- and low-risk groups for Kaplan–Meier analysis to evaluate their correlation with 10-year survival. For each gene in each cancer, Cox proportional hazard regression analysis was used to obtain the Cox coefficients, *P* values (log-rank test), *z* scores, and hazard ratios (HRs) with 95% confidence intervals. A gene with a *P* value of 0.05 or less was considered to have a significant prognostic correlation in each cancer, and genes with HR > 1 (or *z* score > 0) and < 1 (or *z* score < 0) were considered adverse and favorable prognostic genes, respectively (Supplementary Table S8).

### Evaluation of the association between the DEG list (or each of its DEGs) and a specific gene set

**Figure 1.**
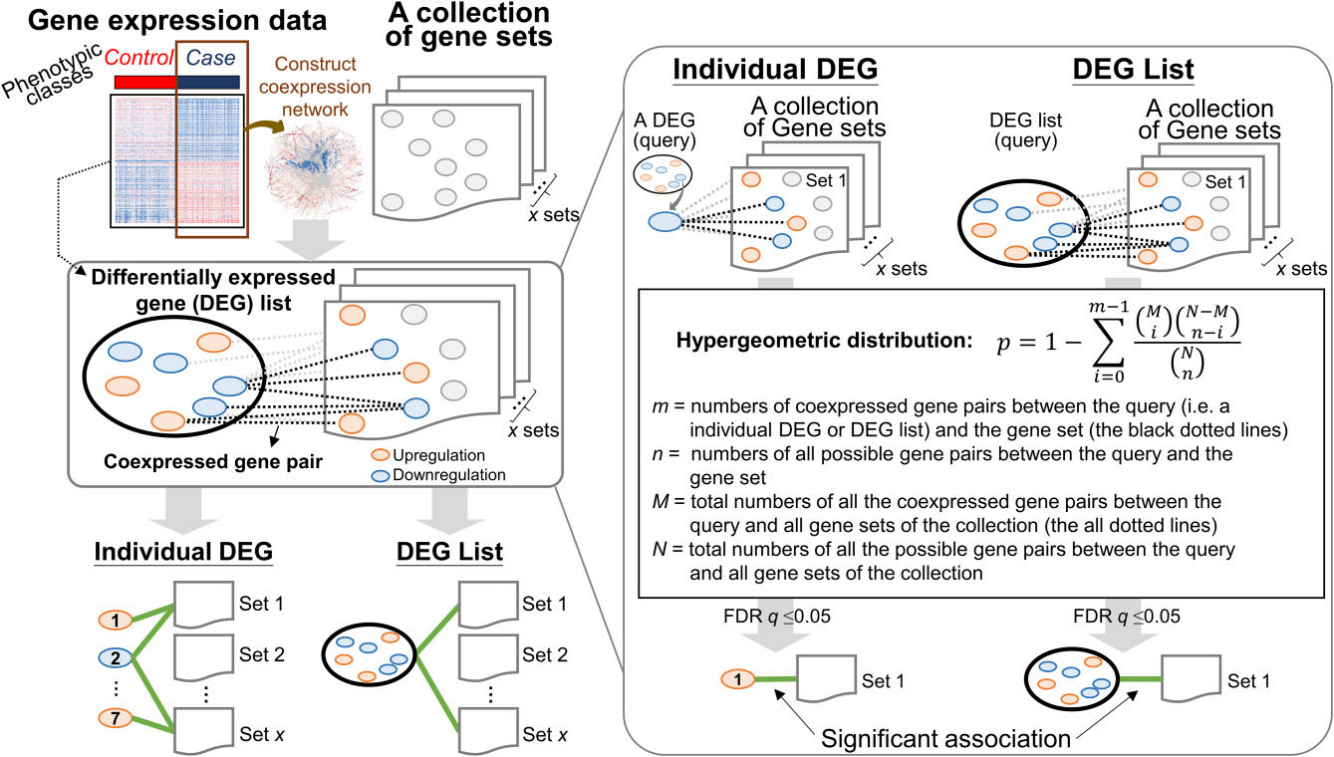
Schematic overview of gene set correlation enrichment analysis (Gscore). First, given expression profiles with two phenotypic classes (i.e. control and case) and a collection of gene sets (e.g. gene sets of all KEGG human pathways), differentially expressed genes (DEGs) were identified by comparing the control samples and case samples. Next, we constructed the coexpression network using the expression profiles of case samples, of which two DEGs (blue or orange circle) with a Pearson correlation coefficient (|Pearson's r|) ≥ *c* (e.g. *c* is 0.3, 0.5, or 0.7) across case samples were considered a coexpressed gene pair (dotted lines). Finally, Gscore used the coexpressed gene pairs between the DEG list (or each of its DEGs) and each gene set in this collection to determine whether the association was statistically significant (FDR *q*value ≤ 0.05) based on the hypergeometric distribution with Benjamini–Hochberg correction.

To measure the statistical significance of associations between a list of selected DEGs and a specific gene set (e.g. a group of genes in a KEGG pathway), the Gscore method evaluates the enrichment of coexpressed gene pairs between all the DEGs of the selected list and all the DEGs in the collection of gene sets (e.g. gene sets for 347 KEGG human pathways) based on the hypergeometric distribution with Benjamini–Hochberg correction (Figure 1). Moreover, for each DEG in the selected list, the goal of identifying its association with a certain gene set is to quantify how it contributes to capturing and describing the biological phenomenon based on the correlations and dependencies between it and the gene set. The process details are as follows:

- For each input gene expression dataset with samples belonging to two classes, we first identified the DEGs between control and case samples. Then, we constructed a coexpression network using the expression profiles of the case samples, in which two DEGs with a Pearson correlation coefficient (|Pearson's *r*|) ≥ *c* across case samples were considered a coexpressed gene pair. Here, *c* can be set by the user, for example, to 0.3 (low), 0.5 (moderate) or 0.7 (high). Note that genes with identical expression values across all ‘case’ samples are ignored when calculating the correlation.
- For each DEG in the query list (Supplementary Figure S2A), Gscore first uses the coexpressed gene pairs between that DEG and all the DEGs of a gene set in the selected collection to determine the association significance for this gene set based on the hypergeometric distribution (35,47,48) as follows:
  (1) \begin{eqnarray*}P = 1 - \sum \limits_{i = 0}^{m - 1} \frac{\left(\begin{array}{c} M\\ i \end{array}\right)\left(\begin{array}{c} {N - M}\\ {n - i} \end{array}\right)}{\left(\begin{array}{c} N\\ n \end{array}\right)}\end{eqnarray*}
  where *m* and *n* are, respectively, the numbers of coexpressed gene pairs and all possible gene pairs between each DEG in the query DEG list and all the DEGs in a specific gene set; for instance, the values for *m* and *n* between DEG *a* and Gene Set A (containing 5 DEGs) in Supplementary Figures S2A are 3 (red dotted lines) and 5, respectively. *M* and *N* are, respectively, the total numbers of all the coexpressed gene pairs and all possible gene pairs between each DEG in the query DEG list and all the DEGs in the gene sets of the selected collection. The FDR *q* value for multiple hypothesis testing with the Benjamini–Hochberg method was used (49), and the false discovery rate was controlled at 5%. Here, the association between the DEG in the query DEG list and a certain gene set was considered statistically significant when its *q* value was ≤ 0.05.
- For the query DEG list, Gscore further measured the statistical significance of association for a specific gene set based on the coexpressed gene pairs between all of the involved DEGs and all the DEGs of this gene set in the selected collection (Supplementary Figure S2B). Then, we computed the *P* value of the hypergeometric distribution as follows:
  (2) \begin{eqnarray*}P = 1 - \sum \limits_{i = 0}^{{m}_g - 1} \frac{\left(\begin{array}{c} {M}_{g}\\ i \end{array}\right)\left(\begin{array}{c} {N}_{g} - {M}_{g}\\ {n}_{g} - {i} \end{array}\right)}{\left(\begin{array}{c}{N}_{g}\\ {n}_{g} \end{array}\right)}\end{eqnarray*}

where ${m}_g$ and ${n}_g$ are, respectively, the numbers of coexpressed gene pairs and all possible gene pairs between all the DEGs of the query list and all the DEGs in a specific gene set; for example, ${m}_g$ and ${n}_g$ between List *i* (including 7 involved DEGs) and Gene Set *A* (containing 5 DEGs) in Supplementary Figure S2B are 6 and 35, respectively. ${M}_g$ and ${N}_g$ are, respectively, the total numbers of all the coexpressed gene pairs and all possible gene pairs between all the DEGs of the query list and all the DEGs of gene sets in the selected collection. Here, the association between the query DEG list and a certain gene set was considered statistically significant when its FDR *q* value was ≤0.05 (Benjamini–Hochberg correction).

Brief introductions to and suggested thresholds for the NEA (29), ROntoTools (27,28), SPIA (23), CePaORA (24,25), CePaGSA (24,25), PADOG (18), GSA (17), GSEA (16), and ORA (hypergeometric test) (5,6,11,12) methods are presented in Supplementary Table S9. For the NEA method, the suggested aggregate network, merged6_and_wir1_HC2 (50), was used as input. It was constructed by merging the higher confidence network ‘FClim_HC2’ with the curated links available from several databases (e.g. CORUM (51), Phosphosite (52), KEGG (37), and MSigDB (53)) and the reverse-engineered network ‘wir1’. The FClim_HC2 and wir1 networks were derived from the FunCoup (54) database and the TCGA glioblastoma expression, methylation, and mutation datasets, respectively.

### Pancancer analysis

To assess the association between each DEG list (or its individual DEGs) and the gene set of a certain pancancer pathway with statistical significance across 16 cancer types (i.e. TCGA RNA-seq datasets), the FDR *q* value (or nominal *P* value) for each significant association was transformed to a *z* score (35). The *z* scores in the 16 cancers were further summarized using Stouffer's unweighted *Z*-transform test (55) (i.e. meta-*z* score). Then, Kendall's τ coefficient (*kendalltau* function in *scipy* package) was used to measure the correlation between the associations with 69 pancancer pathways across 16 cancer types identified by Gscore and the other methods.

For an individual DEG, we wanted to observe pathway-wide and cancer-wide signatures associated with prognostic outcomes based on two issues: (i) which gene is involved in the most pancancer pathways, and what survival outcome is correlated with that gene; and (ii) which association between a gene and pancancer pathway is more likely to be shared by distinct tumor types, and what prognostic outcome is correlated with that gene. Therefore, the significance of the pathway-wide and cancer-wide enrichment of each DEG was evaluated by summarizing the *z* scores in 69 pancancer pathways and 16 distinct cancers using Stouffer's method (unweighted). To determine the pancancer prognostic significance of each gene, we further evaluated the meta-*z* score based on the *z* scores of 10-year survival outcomes in 16 cancer types by Cox proportional hazards regression analysis.

## Results

### Gene set correlation enrichment analysis (Gscore)

Figure 1 illustrates the process of using Gscore to annotate and interpret the DEGs in the RNA expression profiles between two phenotypic classes, i.e. ‘control’ and ‘case’. A DEG list is derived from the profiles of both control and case samples. The coexpression network constructed by using the profiles of case samples is meant to describe and capture the biological phenomenon related to a given disease or condition. Given the DEG list, the coexpression network, and a collection of gene sets (e.g. groups of genes that belong to the same pathway or share a common biological function), Gscore aims to determine whether the DEG list and its individual DEGs are significantly associated with a specific gene set. Based on the hypergeometric distribution and statistical correction, the significance of association between the DEG list (or each individual DEG) and the gene set can be determined (Figure 1 and Supplementary Figure S2; details in Materials and methods). A recent study provided a comprehensive comparison of the performances of 13 pathway analysis methods based on their ability to identify target pathways in 75 human microarray datasets (2). To evaluate and compare the accuracy, robustness, and applicability of our Gscore method with different existing methods, five TB methods (e.g. NEA (29–31), ROntoTools (27,28), SPIA (23), CePaORA, and CePaGSA (24,25)) and four non-TB methods (e.g. PADOG (18), GSEA (16), GSA (17), and ORA (hypergeometric test) (5,6,11,12)) were chosen as representative methods according to their performances in the previous study (2). These methods were implemented by the corresponding tools (e.g. R packages) using the parameters and thresholds suggested by the original authors (Supplementary Table S9). Note that the genome-wide gene expression data (or corresponding DEG lists) and the pathways (or corresponding gene sets) were used as inputs according to the requirements of the different methods.

### Comparison of association detection for DEG lists by different pathway analysis methods

**Figure 2.**
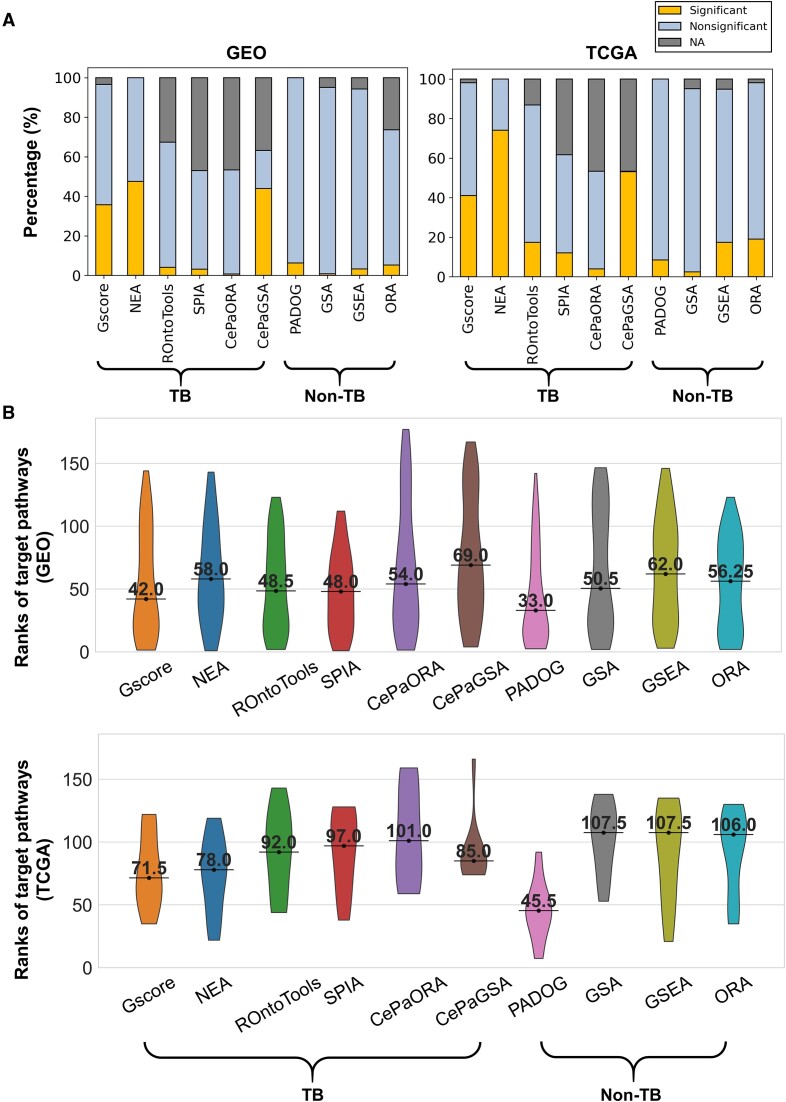
Comparison of six topology-based (TB) and four nontopology-based (non-TB) methods for detecting associations with KEGG pathways or their gene sets. (**A**) Distributions of the percentages of significant and nonsignificant associations between the DEG lists (or expression data) and the 347 KEGG pathways (or their gene sets) identified by ten different methods. For some methods, the DEG lists were separately derived from the 75 microarray expression datasets in 15 disease types assembled from the GEO database (left) and the 16 TCGA RNA-seq datasets in 16 cancer types (right) as inputs. For example, the percentage of significant associations across 16 TCGA RNA-seq datasets is the cumulative number of significant associations (i.e. impacted pathways) divided by the cumulative number of all possible associations ($347 \times 16$). For each method, the significance level threshold suggested in the original work is used (details in Supplementary Table S9). Note that an association was considered not applicable (NA) for the method if its corresponding pathway (or gene set) was filtered out in the preprocessing step or unavailable in the evaluation step. (**B**) Violin plots showing the ranks of target pathways identified by the ten methods using 75 GEO microarray datasets (top) and 10 TCGA RNA-seq datasets (bottom). Among the collected datasets, those relevant to diseases that already have a corresponding KEGG pathway (i.e. target pathway) are used; method performance is assessed according to the ability to rank the target pathway. In each plot, the value represents the median of ranks across the 75 GEO (or 10 TCGA) datasets.

To infer the associations between the DEG lists and the KEGG pathways (or their gene sets) using distinct pathway analysis methods (i.e. six TB and four non-TB methods), we first collected the 75 microarray datasets related to 15 diseases from the GEO database (Supplementary Table S1). Next, we further assembled and curated RNA-seq datasets in 16 cancer types from TCGA (Supplementary Table S2) to independently benchmark these methods on RNA-seq datasets. Then, the DEG list derived from each dataset was obtained as the query list for the methods that require DEG lists as input. The gene sets derived from 347 human pathways were collected from the KEGG database. Based on 75 GEO microarray datasets and 16 TCGA RNA-seq datasets, the average percentages of significant associations with the KEGG pathways (or their gene sets) detected by Gscore (∼38%) were lower than those detected by the NEA (∼61%) and CePaGSA (∼48%) methods and greater than those detected by the other methods (<19%; Figure 2A). Note that the KEGG pathway version used for the CePaORA and CePaGSA methods are unknown because the pathway data were already embedded in the tools, but in both cases the pathway number is smaller than that in our version (v. 102). ROntoTools and SPIA methods are signaling pathway impact analysis methods; thus, some of the 347 pathways are not applicable (NA) for them.

To benchmark these TB and non-TB methods, we first assessed their ability to identify the target pathways using 75 microarray datasets related to 15 diseases and 10 RNA-seq datasets in 10 cancer types. We can expect that a method with better performance would be able to rank the target pathway on top based on its statistical significance (i.e. a small *P* value). Of the TB and non-TB methods, Gscore and PADOG achieved the best performance (i.e. the lowest median rank values), respectively, on two different expression platforms (Figure 2B). This also demonstrates concordance with the results of Nguyen *et al.* (2) showing that PADOG performs better than the other non-TB methods. Additionally, the distributions of median *P* values of the target pathways for different methods are shown in Supplementary Figure S3. We observed that some methods exhibited inconsistent performance in terms of median rank values and *P* values. For example, the CePaGSA method exhibited the highest median rank values but the third-lowest median *P* values in 75 microarray datasets. This is reminiscent of previous reports that the estimate of a method based on the derived *P* values of the target pathways is not as reliable as that based on rank values due to its intrinsic bias of *P* values toward zero (2). Since the use of unknown and older KEGG versions may lead to relatively poor performance, the CePaORA and CePaGSA methods were excluded from the subsequent analyses. Taken together, these results suggest that our Gscore method can identify a moderate number of significant associations and has a better ability to detect target pathways than the other TB and most of the non-TB methods (except for the PADOG method), regardless of the expression platform.

### Pancancer analysis of significant associations

**Figure 3.**
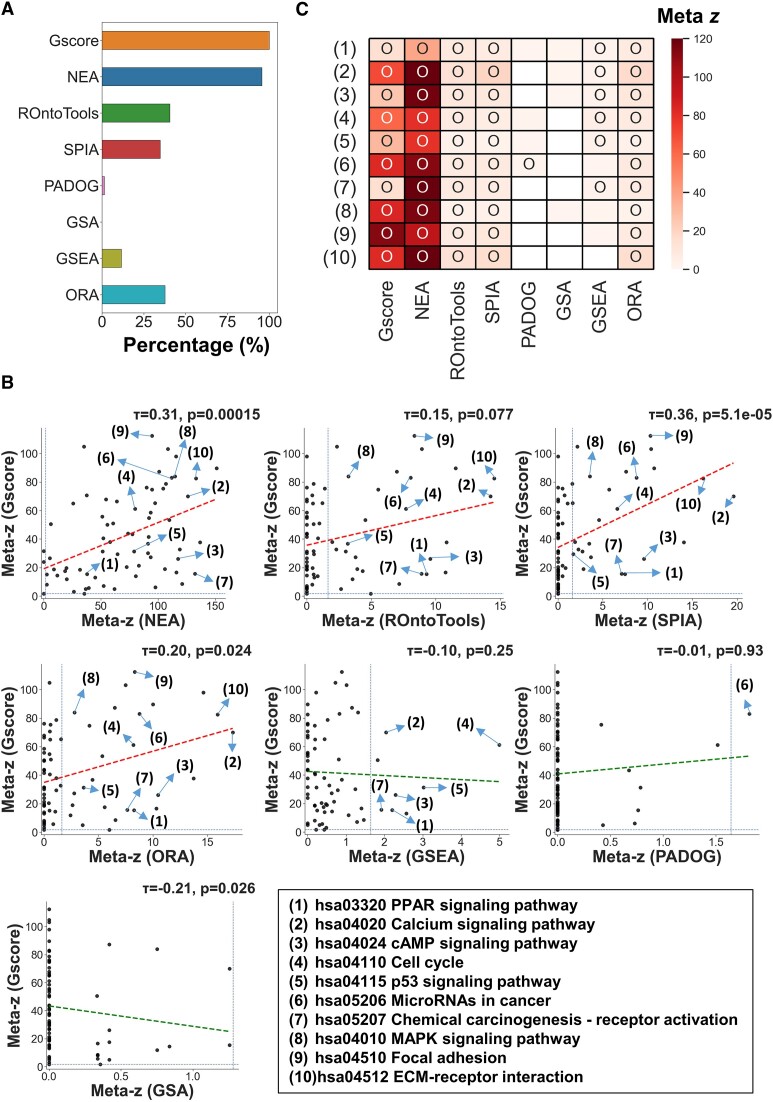
Pancancer analysis of the significant associations between DEG lists and pancancer pathways across 16 cancer types using eight different methods. (**A**) Percentages of significantly impacted pancancer pathways among 69 pancancer pathways detected by different methods. A pancancer pathway was considered significantly impacted across 16 cancers when its meta-z score was ≥ 1.64 (nominal one-sided *P* value < 0.05). (**B**) Meta-*z* scores of associations with pancancer pathways identified by Gscore versus by the other seven methods. The meta-*z* score indicates the statistical significance of an association between the DEG list and each pancancer pathway across 16 cancers. The dots in quadrants I and III represent the significant (meta-z ≥ 1.64) and nonsignificant (meta-z < 1.64) associations across 16 cancers, respectively, identified by Gscore and other methods. Kendall's τ coefficient is used to measure the correspondence in each plot (red: positive correlation; green: negative correlation). (**C**) Heatmap of meta-*z* scores for the top 10 shared pancancer pathways significantly impacted across 16 cancers using eight methods. These top 10 shared pancancer pathways are labeled in (B), in which the number symbols refer to the pathways listed in the bottom-right panel of (B). Circles are used to mark statistically significant associations (meta-z score ≥ 1.64).

The pathway analysis method is valuable in interpreting and annotating gene expression data, especially in cancer research (16,56). Cancer is a consequence of abnormalities in various biological pathways that interact in a complex network (35,56,57); therefore, cancer researchers usually try not only to identify the target pathway of a specific cancer type but also to find any other pathways that are relevant to the target pathway (58,59). For instance, alterations in several signaling pathways that regulate cell growth, cell cycle progression, and apoptosis are common hallmarks of different types of cancers (also called pancancer pathways) (60). Therefore, we assumed that the expression datasets (or the DEG lists derived from them) in cancers are likely to be significantly associated with pancancer pathways. To assess the ability of various pathway analysis methods to identify pancancer pathways on TCGA RNA-seq datasets in 16 cancers, we first collected a total of 69 pathways belonging to either the pathways in the ‘6.1 Cancer: overview’ category or pathways related to the pathways in this category as defined by the KEGG database (Supplementary Figure S1C and Supplementary Table S4; details in Materials and methods). To investigate the roles of significant associations in a cancer-wide landscape, the significant associations across 16 cancer types were summarized using Stouffer's unweighted *Z*-transform test. Among the 69 pancancer pathways (or their corresponding gene sets), Gscore displayed the most efficient ability (∼100%) to identify significantly impacted pancancer pathways, followed by NEA (95.6%) and ROntoTools (41%; Figure 3A). Moreover, the meta-*z* scores of associations with pancancer pathways for Gscore showed a positive correlation with those of each of the TB methods (SPIA (τ = 0.36, *P* = $5.1 \times {10}^{ - 5}$, Kendall's τ coefficient), NEA (τ = 0.31, *P* = 1.5$ \times {10}^{ - 4}$) and ROntoTools (τ = 0.15, *P* = 0.077)) as well as those for the ORA (τ = 0.20, *P* = 0.024) method (Figure 3B), suggesting that they are consistent to some extent.

Figure 3C shows the top 10 shared pancancer pathways significantly impacted across 16 cancers as identified by the eight methods. Some of these signaling pathways are implicated in the development and progression of multiple cancer types, including the MAPK signaling pathway (61), PPAR signaling pathway (62), calcium signaling pathway (63,64), and cAMP signaling pathway (65); other pathways generally relevant to sustaining proliferative signaling (e.g. cell cycle (66)), evading growth suppressors (e.g. p53 signaling (67,68)), and activation of invasion and metastasis (e.g. focal adhesion (69) and ECM-receptor interaction (70)) were identified as common pancancer signatures (68) (Figures 3B and 3C). Notably, the oxidative phosphorylation, basal transcription factors, and Notch signaling pathways play important roles in tumorigenesis (71–73) but were detected only by Gscore (Supplementary Figure S4). For example, previous studies have indicated that the oxidative phosphorylation system is upregulated in many tumors and is involved in tumor progression and aggressiveness at multiple stages of development (71,74). The Notch signaling pathway has been indicated to have a complex relationship with cancer, acting as either an oncogene (e.g. in breast cancer) or a tumor suppressor (e.g. in myeloid malignancy) depending on the specific tissue and cellular context (73). Tumor cells usually exhibit a higher demand for basal transcription than normal cells, and mutations that enhance transcription are frequently found in cancer cells (72). These results are reminiscent of the high cancer-wide concordance reported among genome-wide prognostic genes (75) and membrane protein-regulated pathways (35) and also imply that Gscore can overcome the limitations of the other methods to better identify associations with pancancer pathways.

Next, the joint average relative specificity similarity (joint-AvgRSS) scores (76,77) for GO biological process (BP) and cellular component (CC) terms were used to quantify whether two groups of genes (i.e. the DEG list and gene set of each pathway) are likely to be involved in similar biological processes and located in the same or adjacent cellular components (35,48,78) (Supplementary Note 1). Note that the joint-AvgRSS score could be evaluated only for those methods (i.e. Gscore, NEA, ROntoTools, SPIA, and ORA) that use a DEG list as input. Based on the RNA-seq datasets in 16 cancers and the 69 pancancer pathways, we observed that the significant associations identified by Gscore displayed the highest joint AvgRSS scores, followed by those of the NEA and SPIA methods (Supplementary Figure S5, left). Moreover, the NEA method exhibited the lowest joint-AvgRSS scores for the nonsignificant associations, followed by the Gscore and ROntoTools methods (Supplementary Figure S5, right). These results suggest that two groups of genes determined to have significant (or nonsignificant) associations by the Gscore and NEA methods tend to participate in similar (or distinct) processes and to be localized in the same or adjacent (or distant) cellular compartments.

To further validate the identified associations, we applied the different methods to 45 microarray datasets related to nine shared cancer types corresponding to the TCGA RNA-seq datasets. Using these datasets, the significant associations with the pancancer pathways (or their corresponding gene sets) were first identified using the different methods. We then calculated the overlap coefficient (OC) for significant associations identified using the corresponding TCGA RNA-seq and GEO microarray cancer datasets for each cancer type. The results showed that the median overlap coefficients for the Gscore and NEA methods were higher than 85%, outperforming those for the other methods (<60%; Supplementary Figure S6A). Consistent results were also found for the significant associations between all 347 KEGG pathways and DEG lists (Supplementary Figure S6B), reflecting that the Gscore and NEA methods generate highly reproducible expression profile annotation and interpretation results even when data from different gene expression platforms are used.

### Comparison of association detection for individual DEGs using different pathway analysis methods

**Figure 4.**
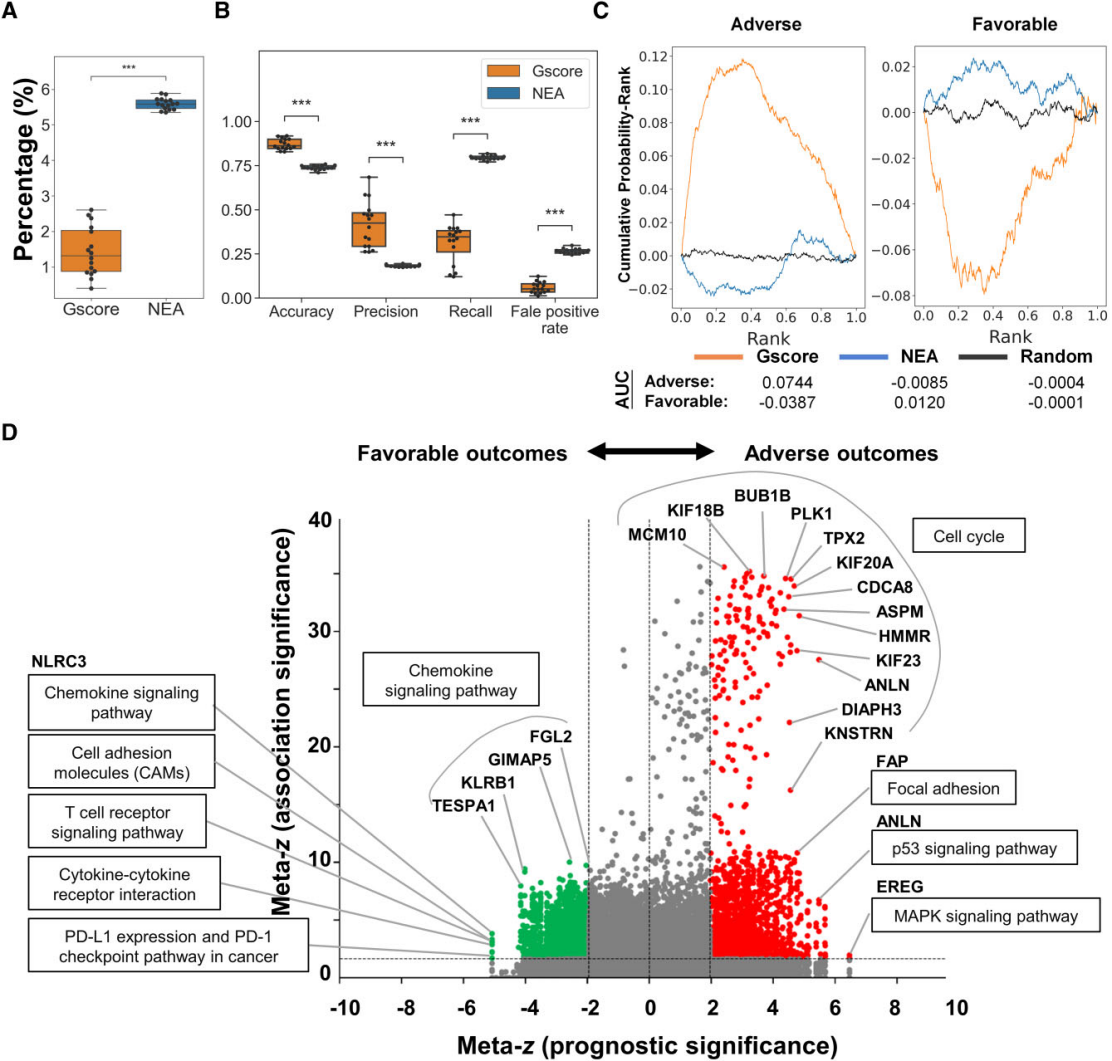
Quantification of Gscore and NEA methods for identifying associations between the individual DEGs and pathways using TCGA RNA-seq datasets in 16 cancers. (**A**) Distributions of the percentages of significant associations among all possible pairs between 347 pathways and all individual DEGs derived from 16 RNA-seq datasets. (**B**) Distributions of the accuracy, precision, recall, and false positive rate (FPR) for detecting the genes involved in 69 pancancer pathways in 16 cancers using the Gscore and NEA methods. Among a total of 3443 genes in 69 pancancer pathways, the DEGs identified from the RNA-seq data in each cancer type were used as query genes. For each pancancer pathway in a cancer type, the DEGs recorded in this pathway (i.e. involved genes) and the remaining DEGs (i.e. genes belonging to other pathways) among the query genes were considered the standard positive and negative cases, respectively. *P* values < 0.001 are indicated by a triple asterisk (Wilcoxon signed-rank test). (**C**) Deviation of the cumulative distribution from uniform (DCDU) for the scaled ranks of adverse (left) and favorable (right) prognostic genes derived from significant associations identified by the Gscore and NEA methods. The rank distribution of randomly ordered prognostic genes is plotted as a black solid line. The AUC is calculated as a measure of the level of deviation from uniformity. (**D**) Volcano plot of the meta-*z* scores of adverse (red: e.g. *KIF18B* gene-impacted cell cycle pathway) and favorable (green: e.g. *NLRC3* gene-impacted chemokine signaling pathway) prognostic genes (*x*-axis) versus the meta-*z* scores of the FDR *q* values for associations between DEGs and pancancer pathways (*y*-axis) across 16 cancer types identified by the Gscore method. The gray dots represent nonsignificant associations (meta-*z* < 1.64 or nominal one-sided *P* > 0.05) and nonsignificant prognostic genes (|meta-z| < 1.96 or nominal two-sided *P* > 0.05).

Among all the DEGs in a list that are significantly associated with a specific pathway, we hypothesized that a DEG is more likely to play a key role in impacting this pathway if it is coexpressed (or interacts) with most of the genes in this pathway but not those in other pathways. In other words, for a specific pathway, the statistical significance of associations for individual DEGs in the list offers an assessment for the prioritization of DEGs based on their impacts on that pathway. To examine whether the pathway analysis methods could distinguish associations for individual DEGs in a list, we applied the different methods to all of the individual DEGs derived from each TCGA RNA-seq dataset. Note that only the Gscore, NEA, ROntoTools, and SPIA methods, which allow querying of an individual gene, were used in this analysis. Similar to the results for the DEG list, the NEA method detected the highest percentages (5.6%) of significant associations among all possible pairs between 347 pathways and all individual DEGs from the 16 RNA-seq datasets, followed by Gscore (1.3%; Figure 4A and Supplementary Figure S7). We further found that the percentages of significant associations for individual DEGs identified by the ROntoTools and SPIA methods were very low (<0.3%; Supplementary Figure S7); thus, only the Gscore and NEA methods were considered in the subsequent analyses.

We next asked whether the DEGs involved in the 69 pancancer pathways could be linked with their original pathways using the Gscore and NEA methods. For each pancancer pathway in a cancer type, the DEGs recorded in this pathway (i.e. involved DEGs) and the remaining DEGs (i.e. DEGs belonging to other pathways) among all the query pathways are considered positive and negative cases, respectively. Here, accuracy, precision, recall, and false positive rate (FPR) were evaluated by using equations S1-S4 in Supplementary Note 2. The results showed that Gscore achieved significantly higher accuracy (median of 0.863) and precision (median of 0.425) and significantly lower recall (median of 0.346) and FPR values (median of 0.051) than the NEA method (*P* values < 0.001, Wilcoxon signed-rank test; Figure 4B). These results suggest that Gscore could efficiently avoid identifying false positive associations but may also miss some positive associations.

To investigate whether DEGs and pancancer pathways exhibited prognostic correlations with human malignancies, we first assessed the correlation of each DEG with 10-year survival outcomes based on gene expression and clinical outcome data from TCGA. Next, we used the meta-*z* scores of associations for individual DEGs across the 69 pancancer pathways to evaluate and rank the significance of their pathway-wide enrichment. The ranks of adverse (left) and favorable (right) prognostic genes were further examined for the Gscore and NEA methods, and their cumulative distributions (40,79) were plotted (Figure 4C and Supplementary Note 3). Gscore displayed higher positive and negative area under the curve (AUC) values for benchmark gene sets with adverse (left) and favorable (right) prognostic outcomes, respectively, than gene sets identified by the NEA method and random gene sets. This finding implies that Gscore could reflect that genes are more likely to be correlated with adverse (or favorable) survival when involved in multiple (or specific) pancancer pathways.

To further examine the relationship between prognostic significance and association significance, we defined the genes involved in significant associations across 16 cancer types (i.e. meta-z ≥ 1.64) as adverse and favorable prognostic genes when their meta-*z* scores of prognostic significance were > 1.96 and < −1.96, respectively. Statistical analysis demonstrated that the proportion of adverse and favorable prognostic genes was higher among the genes involved in the significant associations with the pancancer pathways across 16 cancer types identified by Gscore than among the genes involved in the nonsignificant associations (*P* = $8 \times {10}^{ - 219}$, chi-square test) and superior to those identified by the NEA method (*P* = $3 \times {10}^{ - 53}$; Supplementary Figure S8). Next, we described some of the most significant associations correlated with adverse or favorable outcomes. Numerous genes involved in cell cycle regulation (e.g. *PLK1*, *TPX2*, and *KIF20A*) were correlated with adverse outcomes and had strong association significance (Figure 4D, right). For example, *PLK1* is categorized as an oncogene and is highly overexpressed in various cancer cells, including gastric, breast, and liver cancers (80–82); *TPX2* is also overexpressed in cancers and is considered a diagnostic and prognostic marker for malignancies (83); and *KIF20A* has been observed to be upregulated in and capable of regulating malignant behavior in numerous cancer types (84–86). The expression of *EREG* (encoding proepiregulin), which belongs to the ErbB family of ligands and is involved in the MAPK signaling pathway, was most frequently correlated with adverse outcomes. *EREG* is overexpressed in many human cancers and has been implicated in tumor progression (e.g. resistance to apoptosis) and metastasis (87). Interestingly, the expression of several genes associated with various immune-related pathways (e.g. chemokine signaling pathway, T cell receptor signaling pathway, and PD-L1 expression and PD-1 checkpoint pathway in cancer) exhibited favorable prognostic associations (Figure 4D, left). For instance, the expression of *NLRC3*, which encodes NLR family CARD domain-containing protein 3 and functions as a potential tumor suppressor through the inhibition of cellular proliferation and stem-cell-derived organoid formation (88) and the regulation of inflammation (89) in several pathways (e.g. chemokine signaling), was most frequently correlated with favorable outcomes. In view of the above results, our Gscore method could provide insights for future investigations of how pathway-wide and cancer-wide associations for individual genes reflect prognostic outcomes.

### Annotation and interpretation of gene expression data from cells and patient samples with COVID-19 infection

**Figure 5.**
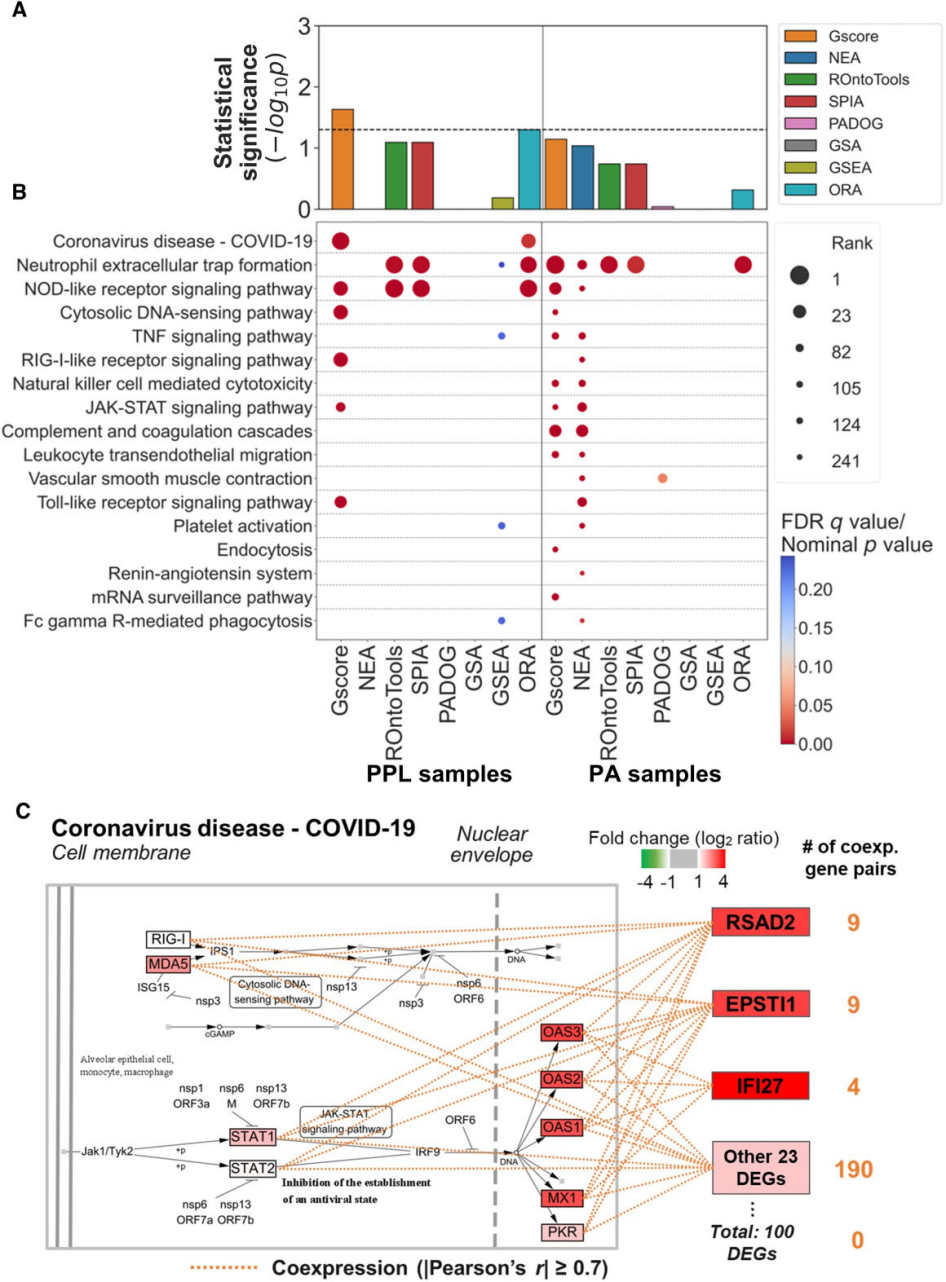
Detection of associations between COVID-19-related pathways and the DEG lists derived from RNA-seq datasets of plasma and leukocyte (PPL) samples and autopsy (PA) samples from patients with or without COVID-19 infection using eight methods. (**A**) Bar chart (top panel) showing the statistical analysis of whether the proportion of COVID-19**-**related pathways was higher among the significantly impacted pathways than among the nonsignificantly impacted pathways for two different DEG lists. The dashed line indicates the statistical significance threshold of *P* value < 0.05 ($ - {\log }_{10}P >1.301$, Fisher's exact test). (**B**) Dot plot showing the significant associations between 17 COVID-19**-**related pathways and the DEG lists derived from two RNA-seq datasets. The dot size is proportional to the rank of each COVID-19-related pathway identified by the corresponding method. The color bar indicates the nominal *P* value for the PADOG method and FDR *q* values for the other methods. (**C**) Association between the coronavirus disease - COVID-19 pathway (hsa05171) and the list of top 100 DEGs derived from the PPL sample dataset detected by Gscore. In this list, 26 of 100 DEGs were coexpressed with at least one DEG in the pathway; the top 3 ranked DEGs (*RSAD2*, *EPSTI1*, and *IFI27*) with the higher expression fold changes and their coexpressed gene pairs are shown. The squares with colored and bold letters represent the DEGs in the list and the pathway. Orange dotted lines indicate the coexpressed gene pairs (|Pearson's *r|* ≥ 0.7) between DEGs in the list and the pathway. In this pathway, the upregulated (red) and downregulated (green) DEGs coexpressed with DEGs in the list and their interacting neighbors are labeled with gene names.

As of 21 June 2023, the World Health Organization (WHO) reported more than 768 million infections and 6.9 million deaths related to COVID-19. It is necessary to understand the disease mechanisms to control the pandemic. To further apply and validate our Gscore and the other methods to this important problem, we first collected four RNA-seq datasets from samples derived from patients with or without COVID-19 (or cells with or without SARS-CoV-2 infection) from the GEO database, including patient-derived plasma and leukocyte (PPL) samples (GSE157103) (41), patient-derived autopsy (PA) samples (GSE150316) (42), human bronchial epithelial (NHBE) cells (GSE147507) (43,44), and alveolar type 2 progenitor (AT2) cells (GSE160435) (45). Additionally, we obtained 17 COVID-19-related pathways and the corresponding gene sets in humans, including the target pathway (i.e. Coronavirus disease—COVID-19, KEGG ID: hsa05171) and its 16 related pathways as defined in the KEGG database (Supplementary Table S7). By applying different pathway analysis methods to each RNA-seq dataset, we detected the associations with all the KEGG human pathways (or their gene sets) to examine whether the COVID-19-related pathways are significantly impacted in a given phenotype. Statistical analysis showed that the proportions of COVID-19-related pathways among the significantly impacted pathways detected by Gscore were higher than those among the nonsignificantly impacted pathways on the datasets of PPL samples (*P* value = 0.023, Fisher's exact test), PA samples (*P* value = 0.072), and NHBE cells (*P* value = $6 \times {10}^{ - 5}$), outperforming the other methods (Figures 5A and B and Supplementary Figures S9A and S9B). On the AT2 cell dataset, the proportions of COVID-19-related pathways achieved statistical significance (*P* value < 0.05 or $ - {\log }_{10}P >1.301$, Fisher's exact test) only when using Gscore, ROntoTools, SPIA and PADOG. Notably, Gscore also achieved the best performance in terms of rank (i.e. the lowest median rank values) for the target pathway ‘coronavirus disease - COVID-19′ in these four datasets, followed by the SPIA and ORA methods (Supplementary Figure S9C).

A recent study demonstrated that patients with COVID-19 had severe symptoms related to hyperinflammation and suggested a total of 32 impacted pathways based on the PA sample and NHBE cell datasets (90). In the cell-derived datasets, the Gscore, NEA, ROnoTools, SPIA, and ORA methods detected ≥ 25 (90.6% for NHBE cells and 78.1% for AT2 cells), 32 (100% and 100%), ≥23 (87.5% and 71.9%), ≥26 (81.3% and 84.4%), and ≥ 25 (90.6% and 78.1%) of the 32 impacted pathways, respectively. In the patient-derived datasets, Gscore identified significant associations with ≥ 15 (46.9%) of the 32 impacted pathways, more than most of the other methods (Supplementary Figure S9D). In short, Gscore is better able to detect not only the target pathway of COVID-19 but also the related pathways in both patient- and cell-derived datasets.

Our Gscore method also revealed possible mechanisms for how DEGs are involved in the target pathway of COVID-19. Among the top 100 DEGs, 26 DEGs had a high correlation (|Pearson's *r* ≥ 0.7|) with those in the target pathway of COVID-19 (Figure 5C), and four of them were directly included in this pathway. Among these 26 DEGs, *RSAD2* (encoding radical *S*-adenosyl methionine domain-containing protein 2) was strongly correlated with several upstream (e.g. *STAT1/2*) and downstream (e.g. *OAS1/2/3* and *MX1*) genes in the interferon-stimulated JAK-STAT signaling pathway (Figure 5C). Previous studies have suggested that the interferon-driven antiviral response in the nasopharynx is triggered by SARS-CoV-2 infection and results in the upregulation of *RSAD2* (an interferon-stimulated gene), which exerts antiviral functions (91,92). Moreover, another two genes, *IFI27* (encoding interferon alpha-inducible protein 27, mitochondrial) and *EPSTI1* (encoding epithelial-stromal interaction protein 1), have numerous strong correlations with the genes in the target pathway of COVID-19. *IFI27* is considered one of the canonical inflammatory signature genes in patients with COVID-19 (93), and transcriptome data revealed the upregulation of *EPSTI1* in bronchoalveolar lavage cells and peripheral blood mononuclear cells from patients with severe COVID-19 (94).

**Figure 6.**
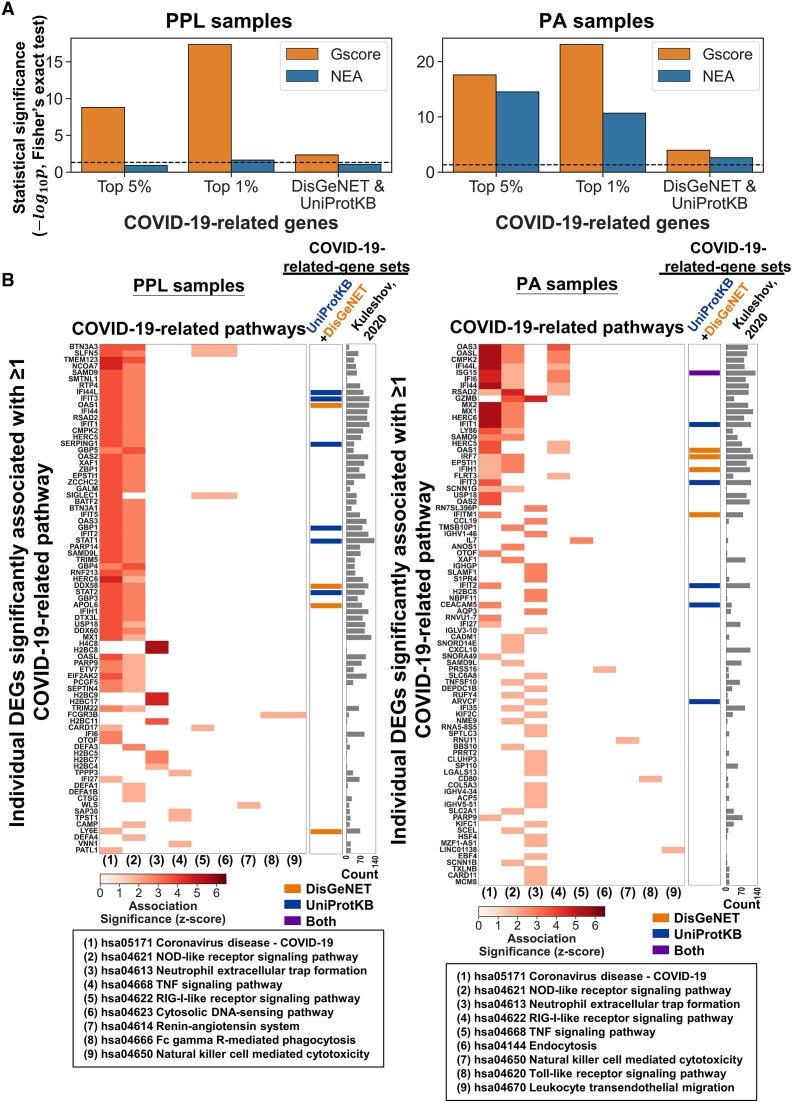
Quantification of Gscore and NEA methods for identifying associations between individual DEGs and COVID-19-related pathways using two RNA-seq datasets (left: PPL samples; right: PA samples) relevant to COVID-19. (**A**) Bar charts showing statistical analyses of whether the proportions of COVID-19-related genes are higher among the DEGs significantly associated with ≥1 COVID-19-related pathway than among the DEGs not significantly associated with any COVID-19-related pathways. From a total of 26162 genes in 722 gene sets in the COVID-19 Drug and Gene Set Library, we derived 923 and 173 COVID-19-related genes with counts within the top 5% and 1% (i.e. most common genes), respectively. Moreover, a merged set consisting of 896 COVID-19-related genes was assembled from the UniProtKB and DisGeNET databases. The dashed line represents the statistical significance threshold of *P* value < 0.05 ($ - {\log }_{10}p >1.301$, Fisher's exact test). (**B**) Heatmaps of significant associations between DEGs and COVID-19-related pathways detected by the Gscore method. The FDR *q* values were transformed into *z* scores to represent the association significance. The COVID-19-related genes recorded in UniProtKB (blue), DisGeNET (orange), or both databases and the COVID-19 Drug and Gene Set Library (the bar chart showing the counts) are marked in the right panels. The number symbols refer to the pathways listed in the corresponding bottom panels.

Additionally, we found that the proportions of COVID-19-related genes among the DEGs associated with ≥1 COVID-19-related pathway were significantly higher than those among the DEGs not associated with any COVID-19-related pathway by the Gscore method, irrespective of clinical sample source (Figure 6A), and Gscore also outperformed the NEA method. Compared to the DEGs with weak associations in specific pathways, the DEGs with strong associations across multiple pathways had higher counts among 722 gene sets collected from the COVID-19 Drug and Gene Set Library (95); furthermore, they were more likely to be included among the COVID-19-related genes assembled from the UniProtKB (96) and DisGeNET (97) databases (Figure 6B). In summary, Gscore using coexpression networks compensates for the limitations of the other methods and provides routes of access for gene expression profile annotation and interpretation of a DEG list of interest and its individual members.

### Implementation of the Gscore method

**Figure 7.**
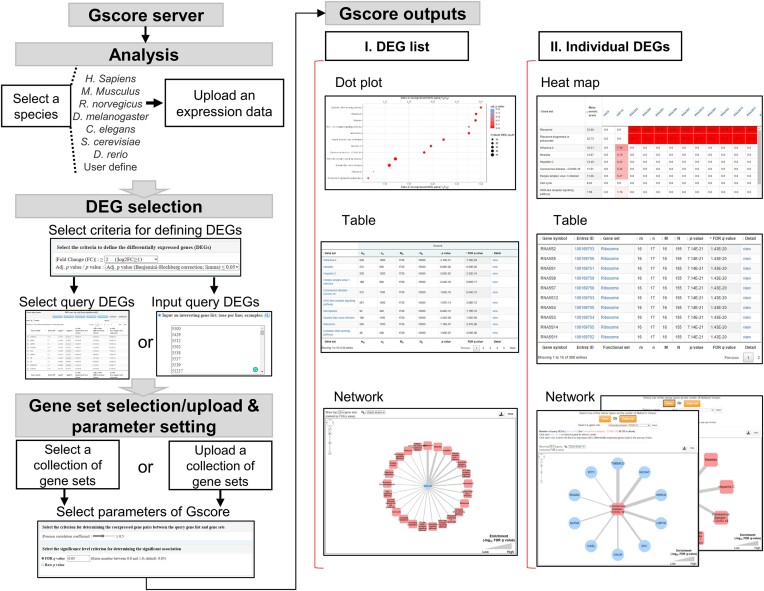
Overview of the Gscore web server (https://gscore.ibsb.nycu.edu.tw/) for annotating and interpreting gene expression data. Users can either upload their own expression profile and gene set data or select curated data collected from several public databases. Gscore offers a user-friendly interface providing a clearly defined procedure, statistically rigorous hypothesis testing, and intuitive visualization of the findings (e.g. interactive tables, dot plots, heatmaps, and network graphs).

To offer Gscore as an online analytical tool, we constructed a server (https://gscore.ibsb.nycu.edu.tw/) through which to interpret and characterize input gene expression data by assessing the statistical significance of the association between the DEG list and a collection of gene sets. The Gscore online tool provides a user-friendly interface with a clearly defined procedure, statistically rigorous hypothesis testing, and intuitive visualization of the findings (Figure 7). Users can first upload their own expression dataset and then either upload a collection of gene sets in several organisms (i.e. *Homo sapiens*, *Mus musculus*, *Rattus norvegicus*, *Drosophila melanogaster*, *Caenorhabditis elegans*, *Saccharomyces cerevisiae* or *Danio rerio*) or select from curated gene sets collected from public databases (37,51,53,77,98–103). Similar to many tools that employ pathway analysis methods, Gscore produces heatmaps, dot plots, network graphs, and tables as output for the visualization of significant associations and coexpressed gene pairs. These output data are available to download for further analysis. An Apache HTTP server running on an Ubuntu system was built for the Gscore online tool, and the webpage was developed using PHP and JavaScript. The Gscore online tool is compatible with the most common web browsers, including Windows Edge, Google Chrome, Mozilla Firefox, and Safari, with no additional plugin installation required. Cytoscape.js (104) is employed for network visualization and also runs without any extra plugins.

## Discussion

Gaining biological insight from genome-wide expression profiles remains a major challenge, and our Gscore method represents a powerful analytical approach for complementing current pathway analysis methods, delineating the relationships between DEG lists (or their individual DEGs) and gene sets defined by prior biological knowledge and aiding in the interpretation of gene expression data.

Gscore has unique advantages over related methods and tools (5,6,11,12,16–18,23–25,27–29). First, using 75 benchmark microarray datasets related to 15 diseases and 10 TCGA RNA-seq datasets in 10 cancer types, Gscore performed better than the other TB methods and most non-TB methods, except PADOG, in identifying the target pathways as significantly impacted (or associated) and ranking them at the top of the impacted pathway list (Figure 2B). Moreover, Gscore achieved the best performance in ranking the target pathway (i.e. coronavirus disease - COVID-19) in four COVID-19 RNA-seq datasets, followed by another TB method, SPIA (Supplementary Figure S9C). These results are consistent with previous findings (1,2,105) that TB methods considering the dependencies and interactions between genes (or proteins) are better able than non-TB methods to detect significantly impacted pathways for further annotating and interpreting gene expression profiles.

Second, Gscore showed good detection ability and reliable results because it does not require users to input a reference network and is not restricted to pathway data formatted in the manner specified by certain databases, which are limitations of the other TB methods. For example, the commonly used human protein–protein interaction (PPI) network from the Search Tool for the Retrieval of Interacting Genes/Proteins (STRING) database (106) is one of the suggested reference networks in EviNet (30), the web tool for the NEA method. However, previous work suggested that this network is inappropriate for cancer data analysis since it includes data from homologous genes in prokaryotic organisms (50). It can be expected that the selection of appropriate reference networks for different research fields will become a challenging issue for users. Additionally, most pathway knowledge bases and PPI networks are constructed by curating the results of experiments conducted in distinct cell types at different time points under diverse conditions; thus, they are considered aggregate (1,40). In general, aggregate networks require frequent updates and integration to be applicable to diverse research topics; specifically, they may fail to promptly include interactions on specific conditions and cells when there is a lack of corresponding experimental data (1), such as emerging infectious diseases like COVID-19. Due to the condition-specific and cell-specific nature of gene expression profiles, the true pathway/network topology depends on the condition being studied and the cell type (1,33). The use of a coexpression network and query DEG list derived from the same gene expression data could offer a way to resolve the above issues (32,34,35,107,108). Thus, we further compared the performances of Gscore when using the dataset-derived coexpression network and the aggregate network, merged6_and_wir1_HC2, based on the ability to identify target pathways in 50 microarray datasets related to 10 cancer types, 25 microarray datasets relevant to 5 other diseases, and 4 COVID-19 RNA-seq datasets. Notably, the merged6_and_wir1_HC2 network includes the wir1 network, which has been suggested as beneficial for cancer data analysis (50). Compared to using the dataset-derived coexpression network, we found that, as expected, Gscore achieved a better performance in cancer-related datasets but worse in datasets related to 5 other diseases and COVID-19 when using the merged6_and_wir1_HC2 network as the reference network (Supplementary Figure S10). Next, to examine whether Gscore performs better when using the dataset-derived coexpression network than when using the aggregate coexpression network, we collected an aggregate coexpression network built by using the microarray and RNA-seq data of 245698 samples from the COXPRESdb database (ver 8.1) (109). By evaluating performance based on the ability to identify target pathways, we observed that Gscore performed better on both the microarray and RNA-seq datasets when using the dataset-derived coexpression network than when using the aggregate coexpression network (Supplementary Figure S11). These results suggest that the Gscore using the dataset-derived coexpression network offers an alternative and promising solution to address the challenges of reference network selection and data integration before obtaining the complete biological network (i.e. interactome).

Third, although prior knowledge about gene characteristics is available through public online databases (e.g. GO (77) and GeneCards (110)), and many pathway analysis methods have been developed (Supplementary Table S9), interpreting a long list of DEGs and the individual DEGs within the list is still a cumbersome and challenging task (111). Network topology in the context of gene or protein interactions allows researchers to examine how individual elements impact larger functional units, enabling the identification of key genes (or proteins) that may play critical roles in biological processes or even diseases (57). Therefore, one of our aims in developing the Gscore method was to enable the detection of specific gene sets significantly associated with the DEG list and each of its DEGs. For example, we observed that compared with the NEA method, Gscore had higher accuracy and precision and lower false positive rate and lower recall in distinguishing the significant associations between 69 pancancer pathways and their involved DEGs (Figure 4B). In contrast, the NEA method is suggested for use for researchers who would like to find as many true associations as possible (i.e. high recall). In addition, when applied to assess the RNA-seq data of patients with COVID-19 (or cells with SARS-CoV-2 infection), we also found that Gscore could comprehensively associate DEG lists with COVID-19-related pathways (Figures 5A and B and Supplementary Figures S9A and S9B) and further determine which of these DEGs are more likely to be COVID-19-related genes (Figure 6).

Fourth, multiple RNA-seq and microarray datasets for human cancers were included to examine the performance and validate the reproducibility of the Gscore method. The overlap analysis for the significant associations identified using the TCGA RNA-seq and GEO microarray datasets in nine cancer types showed highly robust detection ability for the Gscore and NEA methods (Supplementary Figure S6). We further found that the NEA method inferred the highest percentages of significant associations from both RNA-seq and GEO microarray datasets among the methods tested (Figure 2A); this may explain why NEA exhibited the highest overlap coefficients. Among all the methods, NEA achieved the lowest median *P* values (<$6.6 \times {10}^{ - 13}$), followed by CePaGSA (median *P* values = 0.001; Supplementary Figure S3). These results are reminiscent of those of a previous study that reported that a method is likely to generate low *P* values even when the pathway is not significantly impacted if the null distribution of *P* values for this pathway yielded by a method is biased toward zero (2,112).

Finally, integrating Gscore with a meta-*z* score approach across numerous malignancies not only facilitates pancancer analysis to reveal which DEGs and associated pathways are common tumor hallmarks (Figure 4D) but also uncovers important pancancer pathways that the other methods may miss (Figure 3B and Supplementary Figure S4). Our Gscore method further offers guidance for observing how pathway-wide and cancer-wide associations for individual genes reflect clinical outcomes, such as alterations in prognostic correlations (Figures 4C and D). For example, genes involved in multiple and specific pancancer pathways are frequently correlated with adverse and favorable survival outcomes, respectively. The identification of cell cycle associated with many DEGs, including *PLK1*, *ANLN* and *KIF18B*/*20A*, across human cancers demonstrates that the most essential characteristic of cancer cells is sustained proliferation, and that this may lead to adverse survival outcomes (68). Conversely, several tumor suppressors (e.g. *NLRC3* (88)) associated with immune-relevant pathways (e.g. chemokine signaling and T-cell receptor signaling) were found to be the most frequently correlated with favorable outcomes in certain cancers, reflecting the roles of the immune system in antagonizing tumor development and progression (68,113).

Gscore has several limitations, challenges, and perspectives. First, to evaluate the coexpressed gene pairs across samples of the case class and determine the DEGs between two classes via statistically rigorous hypothesis testing, the sample sizes of the control and case classes should be at least three and five, respectively. To examine the influence of sample size of gene expression data on the Gscore method, we utilized 16 TCGA RNA-seq datasets and 75 GEO microarray datasets to execute simulation analysis to detect significant associations based on the gene expression profiles across all tumor samples and across randomly selected samples of different sizes. The results suggested that Gscore performed significantly better when using at least five samples (versus only three samples) in the case class (*P* value $ \le 9.7 \times {10}^{ - 5}$, Mann–Whitney *U* test; Supplementary Figure S12). We believe that the continuous progress in single-cell sequencing and next-generation sequencing technologies (114,115) will offer expression data from large numbers of samples to allow this limitation to be overcome and concomitantly increase the need for data interpretation methods. A second potential constraint of Gscore is that our method may neglect groups of genes that have causal relationships but are not strongly coexpressed because coexpression is less evident in cascade signal transduction, which is usually hierarchical in nature (35). Third, Gscore does not consider the node degree in the gene coexpression network. Indeed, node degrees in the network allow the identification of hub genes that are highly connected with other nodes and potentially represent key regulators or drivers in a specific pathway or even phenotype (116). Nevertheless, a major challenge remains in considering node degree, namely, that this approach can be sensitive to false positive edges if the underlying coexpression network contains spurious or incorrect connections, which may be driven by confounding variables (117); thus, the inclusion of node degree can be expected to affect the reliability of the results. However, Gscore can be further improved in the future by considering the node degrees after reducing false edge discovery in the coexpression network, for example, by using partial correlation or conditional mutual information instead of Pearson correlation coefficient. Finally, another challenge of the Gscore online tool is that it is time-consuming since Gscore must construct a coexpression network for every input expression dataset and simultaneously identify the impacted gene set for each DEG and the entire DEG list. Even so, by following the well-defined procedure provided with the Gscore online tool, users can obtain analysis outputs with intuitive visualizations within several minutes on average.

In conclusion, our results illuminate a strategy for extracting insights from gene expression data and provide numerous clues for further investigation and clinical translation for application in cancer and COVID-19 research. Our Gscore approach also addresses some of the limitations of other widely used methods to further improve the annotation and characterization of high-throughput expression data. Moreover, our Gscore online tool provides an easy-to-use framework to facilitate the annotation and characterization of individual DEGs, DEG lists, and genome-wide expression profiles based on existing biological knowledge.

## Supplementary Material

gkad1187_supplemental_file

## Data Availability

The Gscore online tool is available at https://gscore.ibsb.nycu.edu.tw/. This online tool is free and open to all users and there is no login requirement. Gscore and the code used for analysis are implemented in Python and available at https://github.com/SysMednet/Gscore (permanent DOI: https://doi.org/10.5281/zenodo.8260953). The accession numbers of the gene expression datasets assembled from TCGA and GEO databases are listed in Supplementary Tables S1, S2 and S3.
