# Supplementary material for: Gene set correlation enrichment analysis for interpreting and annotating gene expression profiles
Source: Nucleic Acids Res. 2023 Dec 14;52(3):e17. doi: 10.1093/nar/gkad1187 (PMC10853793; doi:10.1093/nar/gkad1187)
Supplement: gkad1187_supplemental_file [file gkad1187_supplemental_file.pdf]

Gene set correlation enrichment analysis for interpreting and annotating gene expression profiles

Supplementary Data

Lan-Yun Chang[1,†], Meng-Zhan Lee[1,†], Yujia Wu[1,†], Wen-Kai Lee[1], Chia-Liang Ma[1], Jun-Mao Chang[1], Ciao-Wen Chen[1], Tzu-Chun Huang[1], Chia-Hwa Lee[2,3,4,5], Jih-Chin Lee[6], Yu-Yao Tseng[7], and Chun-Yu Lin[1,3,8,9,10,11,*]

[1] Institute of Bioinformatics and Systems Biology, National Yang Ming Chiao Tung University, Hsinchu 300, Taiwan

[2] School of Medical Laboratory Science and Biotechnology, College of Medical Science and Technology, Taipei Medical University, New Taipei City 235, Taiwan

[3] Center for Intelligent Drug Systems and Smart Bio-devices (IDS^{2}B), National Yang Ming Chiao Tung University, Hsinchu 300, Taiwan

[4] TMU Research Center of Cancer Translational Medicine, Taipei Medical University, Taipei 110, Taiwan

[5] Ph.D. Program in Medicine Biotechnology, College of Medicine, Taipei Medical University, Taipei 110, Taiwan

[6] Department of Otolaryngology-Head and Neck Surgery, Tri-Service General Hospital, National Defense Medical Center, Taipei 110, Taiwan

[7] Department of Food Science, Nutrition, and Nutraceutical Biotechnology, Shih Chien University, Taipei 104, Taiwan

[8] Department of Biological Science and Technology, National Yang Ming Chiao Tung University, Hsinchu 300, Taiwan

[9] Cancer and Immunology Research Center, National Yang Ming Chiao Tung University, Taipei 112 Taiwan

[10] Institute of Data Science and Engineering, National Yang Ming Chiao Tung University, Hsinchu 300, Taiwan

[11] School of Dentistry, Kaohsiung Medical University, Kaohsiung 807, Taiwan

[†] The authors wish it to be known that, in their opinion, the first three authors should be regarded as Joint First Authors.

* To whom correspondence should be addressed. Tel: 886-3-571-2121 ext. 59735; Fax: +886-3-5729288; Email: chunyulin@nycu.edu.tw

Supplementary Note 1. Joint average relative specificity similarity (joint-AvgRSS)

To assess whether a DEG list and gene set shared specific biological functions, we first computed the relative specificity similarity (RSS) scores (1,2) of the GO biological process (RSS_{BP}) and cellular component (RSS_{CC}) for each gene pair between the DEG list and gene set (e.g., a group of genes in a KEGG pathway). Next, the mean RSS_{BP} score (or RSS_{CC}), called $AvgRSS_{BP}$ (or $AvgRSS_{CC}$), was measured across all gene pairs between the DEG list and pathway. Finally, the joint-AvgRSS score was evaluated based on their geometric mean, $\sqrt{\mathrm{AvgRSS}_{BP} \times \mathrm{AvgRSS}_{CC}}$. A high joint-AvgRSS score suggests that the genes/proteins are likely to be involved in similar biological processes and located in the same or adjacent cellular components (3-5). Note that the joint-AvgRSS score can be evaluated only for those methods (i.e., Gscore, NEA, ROntoTools, SPIA, and ORA) that use a DEG list as input.

Supplementary Note 2. Comparison of accuracy, precision, recall, and false-positive rates (FPRs) of Gscore and NEA methods for detecting the involved genes of 69 pancancer pathways in 16 cancers

Among a total of 3,443 genes in 69 pancancer pathways, the DEGs identified from the TCGA RNA-seq data in each cancer type were used as query genes. For each pancancer pathway in a cancer type, the DEGs recorded in this pathway (i.e., involved genes) and the remaining DEGs (i.e., genes belonging to other pathways) among all the query genes were considered positive and negative cases, respectively. Thus, significant associations (i.e., FDR q value ≤0.05) were defined as true positive (TP) and false positive (FP) associations when their DEGs are or are not an involved gene in the corresponding pancancer pathway, respectively. The true negative (TN; or false negative, FN) association is a nonsignificant one (i.e., FDR q value >0.05) containing the DEG not recorded (or recorded) in the pancancer pathway. Here, accuracy, precision, recall, and false positive rate are given as follows:

$$Accuracy = \frac{TP + TN}{TP + TN + FP + FN} \quad \text{(S1)}$$

$$Precision = \frac{TP}{TP + FP} \quad \text{(S2)}$$

$$Recall = \frac{TP}{TP + FN} \quad \text{(S3)}$$

$$False\ positive\ rate\ (FPR) = \frac{FP}{FP + TN} \quad \text{(S4)}$$

Supplementary Note 3. Comparison of the performance of the Gscore and NEA methods in the detection of adverse and favorable prognostic genes using the deviation of the cumulative distribution from uniform (DCDU)

To examine the correlation between the pathway-wide enrichment of individual DEGs and adverse or favorable prognostic outcomes in 16 cancers, we used the cumulative distribution function to determine the area under the curve (AUC) scores (6,7) for 16 TCGA RNA-seq datasets to assess and display the distributions of the meta-*z* score rankings of the individual DEGs associated with 68 pancancer pathways. According to the Gscore and NEA methods, the DEG *j* from each TCGA RNA-seq dataset *i* was used to identify the significant associations of the gene sets for 347 KEGG human pathways. A significant association between the DEG *j* and the gene set of a certain pathway for the Gscore and NEA methods was determined when its FDR *q* value was ≤0.05. The FDR *q* value for each significant association was transformed to a z score (4). Then, the significance of the pathway-wide enrichment of DEG *j* was evaluated by summarizing the *z* scores in 69 pancancer pathways using Stouffer's method (unweighted). The rankings for the meta-z score of each DEG are scaled by the total number of DEGs A_i in every dataset *i* to give R_{ij}, i.e., the scaled rank of DEG *j* in dataset *i*; for example, the DEG with the highest (or lowest) meta-z score for a certain dataset *i* is denoted by a value of R_{ij} = 0 (or R_{ij} = 1). For each TCGA RNA-seq dataset *i*, the corresponding benchmark subsets B_i are defined by prior biological knowledge, such as the adverse or favorable prognostic genes. Among all A_i, the rankings of the benchmark subsets B_i are examined as T,

$$T = \bigcup_{i \in G, q \in B_i} R_{iq} \qquad \text{(S5)}$$

where the set of rank values of subsets B_i corresponding to dataset *i* are identified for a total of *G* datasets; for instance, *G* is 16 for the RNA-seq datasets. If the gene sets for benchmark subsets B_i are randomly distributed throughout the ranked lists, we can expect the cumulative distribution function of T, called $C(R)$, across multiple RNA-seq datasets to be a uniform distribution, as

$$C(R) = R \qquad \text{(S6)}$$

Similar to the Kolmogorov–Smirnov test, any significant deviation from a uniform distribution denotes that the benchmark subsets are likely to display high (or low) meta-z scores. Hence, the $C(R) - R$ for significant deviations from zero can be used to quantitatively assess different methods. Compared to a uniform random distribution, a positive (or negative) AUC value indicates that the genes in benchmark subsets B_i are focused at the smaller (or larger) scaled ranks and therefore have greater significance. In other words, the AUC value for measuring the deviation from uniformity is equal to the original AUC value (i.e., the observed cumulative distribution) minus 0.5 (i.e., a perfectly uniform distribution).

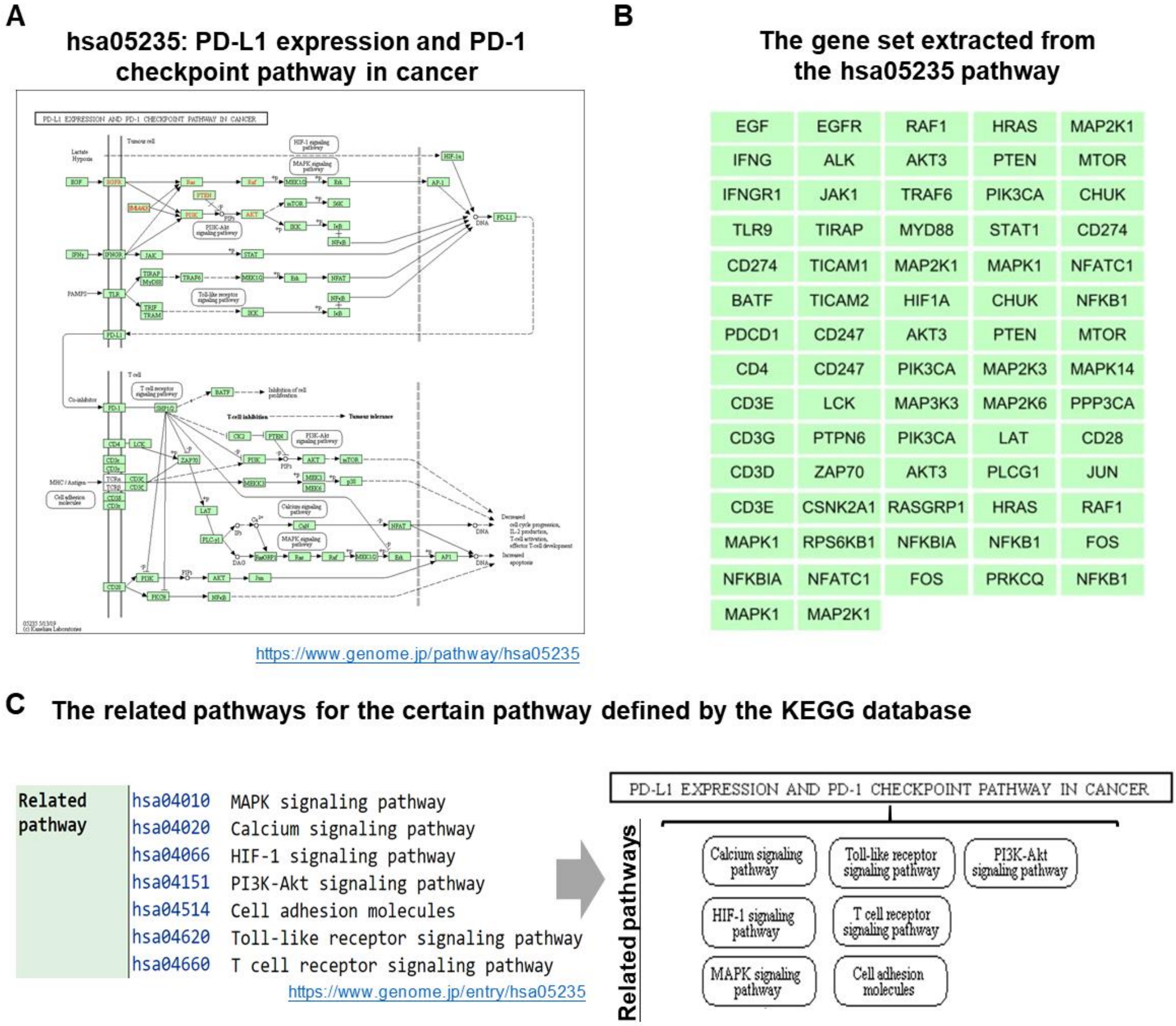

Supplementary Figure S1. Schematic diagram for extracting the gene set and the related pathway(s) from the specific KEGG pathway. (A) PD-L1 expression and PD-1 checkpoint pathway in cancer (hsa05235) from the KEGG database. A pathway shows various genes, the interactions among these genes, the direction of the signal transduction, and other pathways that are related to the original pathway. The image was downloaded from the KEGG database. **(B)** The gene set includes all the genes (without interactions) involved in the original pathway. In other words, the gene set is extracted from the original pathway and stripped of the structure and other additional information. **(C)** Related pathways defined by the KEGG database. For each pathway, related pathways are recorded on its entry page, if there are any related pathways. For example, seven related pathways are recorded for “PD-L1 expression and PD-1 checkpoint pathway in cancer” on its entry page (https://www.genome.jp/entry/hsa05235).

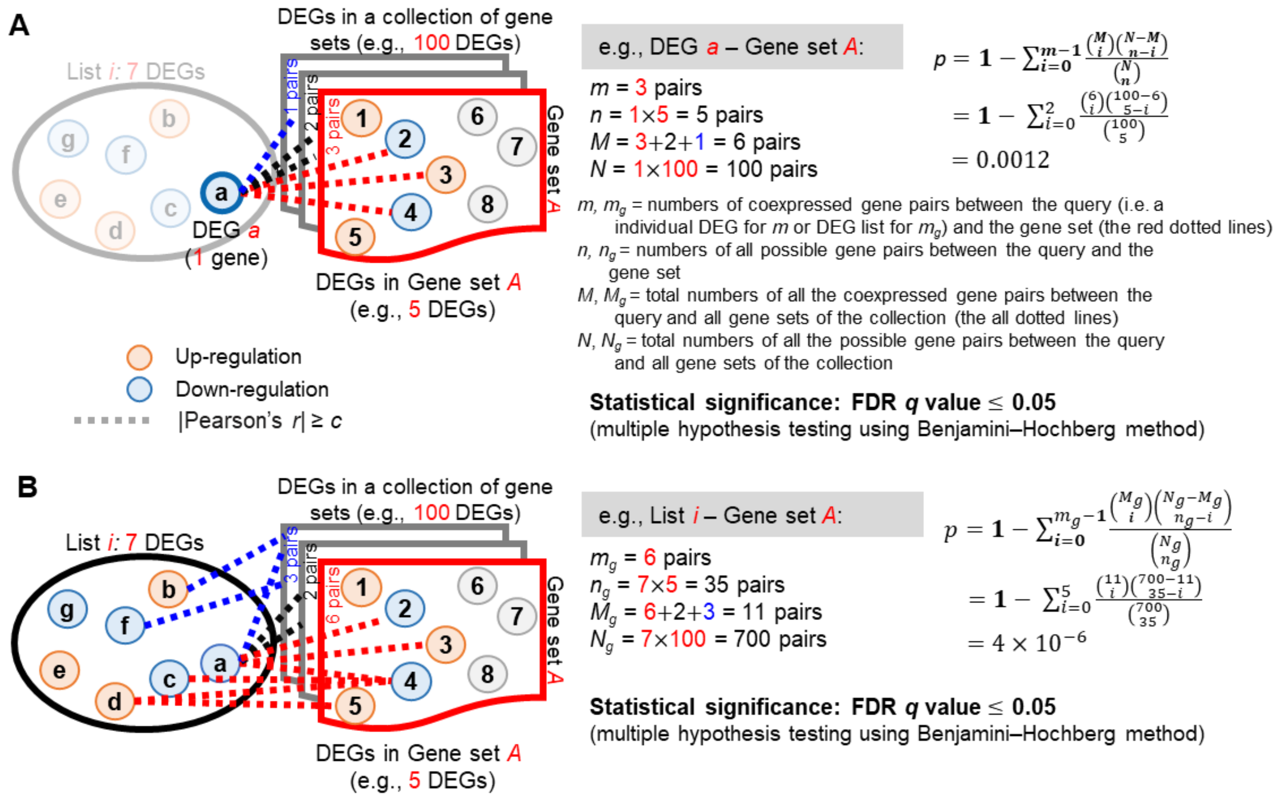

Supplementary Figure S2. Statistical significance evaluation of coexpressed gene pairs between a query DEG list (or each of its DEGs) and a gene set using the hypergeometric distribution. (A) Schematic diagram for calculating the FDR *q*-value of the association between each DEG and the gene set of interest in a collection. Two DEGs with a Pearson correlation coefficient (|Pearson's *r*|) ≥ *c* across case samples were considered to be a coexpressed gene pair (dotted line). Here, *c* can be set by the user, for example, as 0.3 (low), 0.5 (moderate), or 0.7 (high). In the demonstration case, we observed two coexpressed gene pairs (*m*; red dotted lines) and five possible gene pairs (*n*) between DEG *a* and gene set *A,* and six coexpressed gene pairs (*M*; all dotted lines) and 100 possible gene pairs (*N*) between DEG *a* and all the gene sets in this collection. Based on hypergeometric distribution, we can measure the statistical significance (i.e., *p* value) of the association using the *m*, *n*, *M*, and *N* values. Correction for multiple hypothesis testing was performed with the Benjamini–Hochberg method, and the false discovery rate was controlled at 5%. **(B)** Schematic diagram for calculating the FDR *q*-value of the association between the query DEG list, including 7 DEGs, and the gene set of interest in a collection. Similar to the evaluation procedure for each DEG in list *i*, the m_g, n_g, M_g, and N_g values between list *i* and gene set *A* were calculated to measure statistical significance using hypergeometric distribution with Benjamini–Hochberg correction.

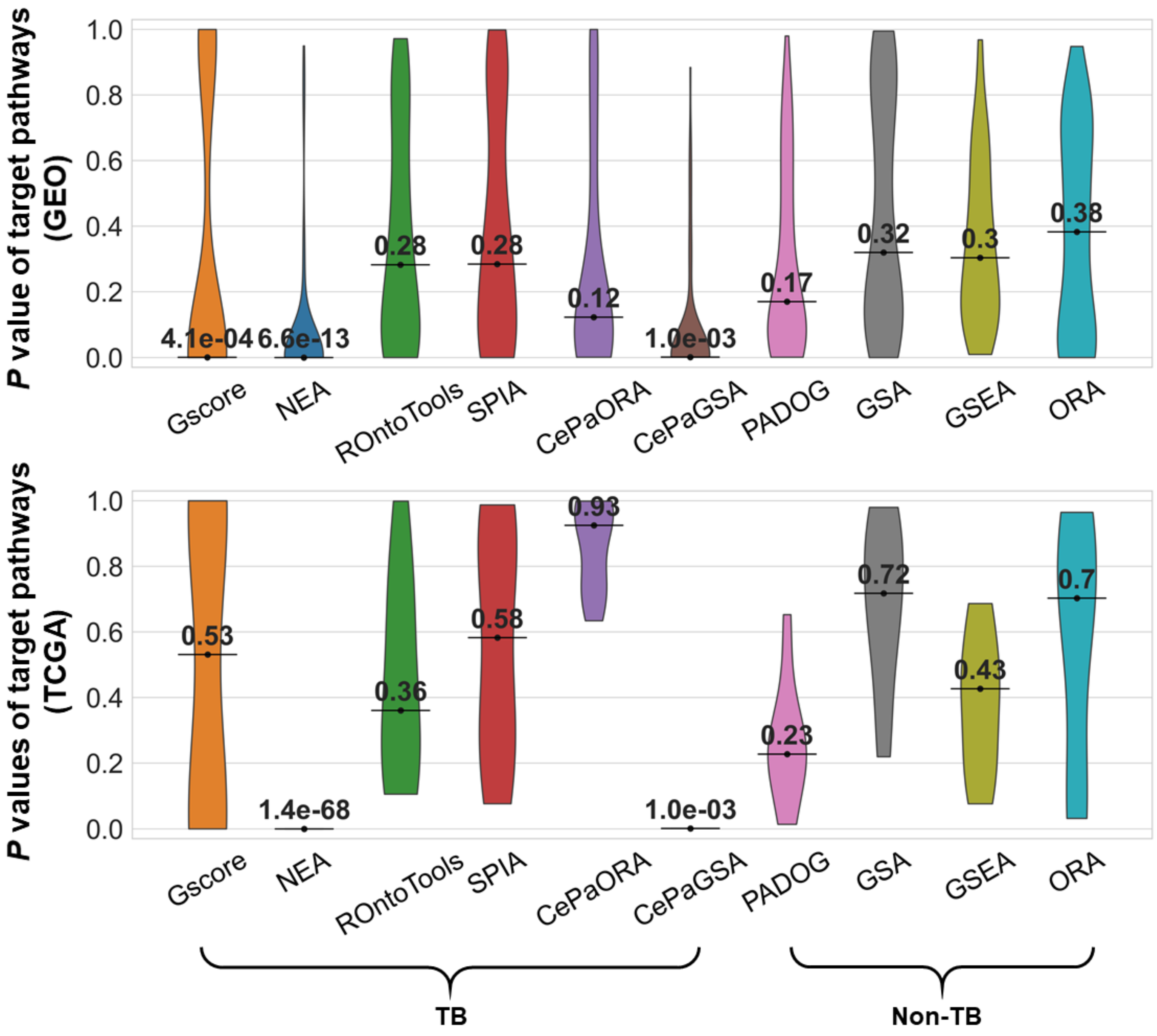

Supplementary Figure S3. Violin plots showing the *p* values of the target pathways identified by six topology-based and four nontopology-based methods in 75 GEO microarray datasets (top) and 10 TCGA RNA-seq datasets (bottom). Among the collected datasets, those relevant to diseases that already have a corresponding KEGG pathway (i.e., target pathway) were used. In each plot, the value represents the median of *p* values across the 75 GEO (or 10 TCGA) datasets.

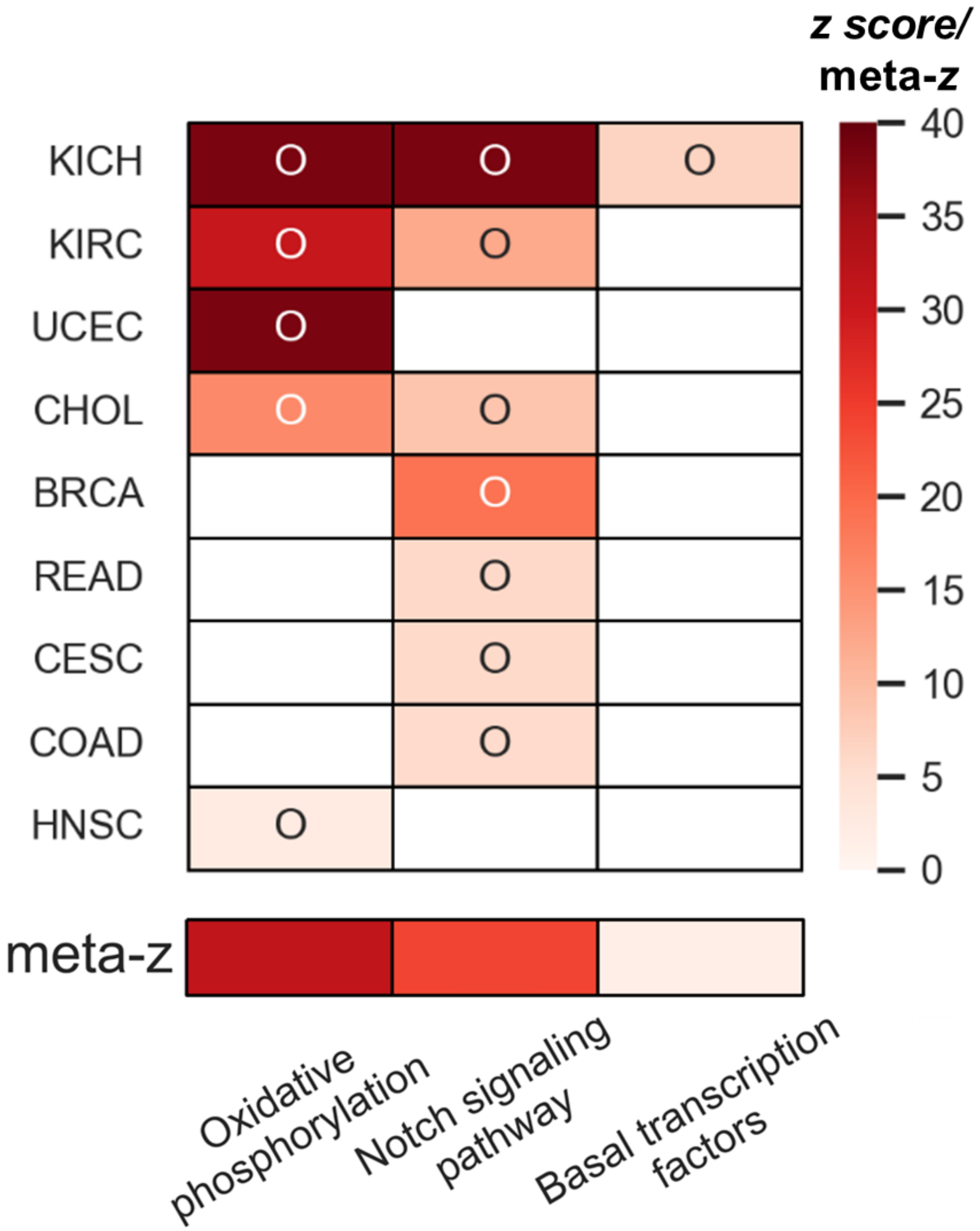

Supplementary Figure S4. Heatmap of *z* scores/meta-*z* scores for the significant associations with the pathways of oxidative phosphorylation, Notch signaling, and basal transcription factors only identified by Gscore in specific cancers. The FDR *q* values were transformed into *z* scores to represent the association significance, in which circles are used to indicate statistically significant associations (FDR $q > 0.05$ or *z* score ≥1.64). The meta-*z* score reflects the statistical significance of an association between the DEG list and each pancancer pathway across 16 cancers.

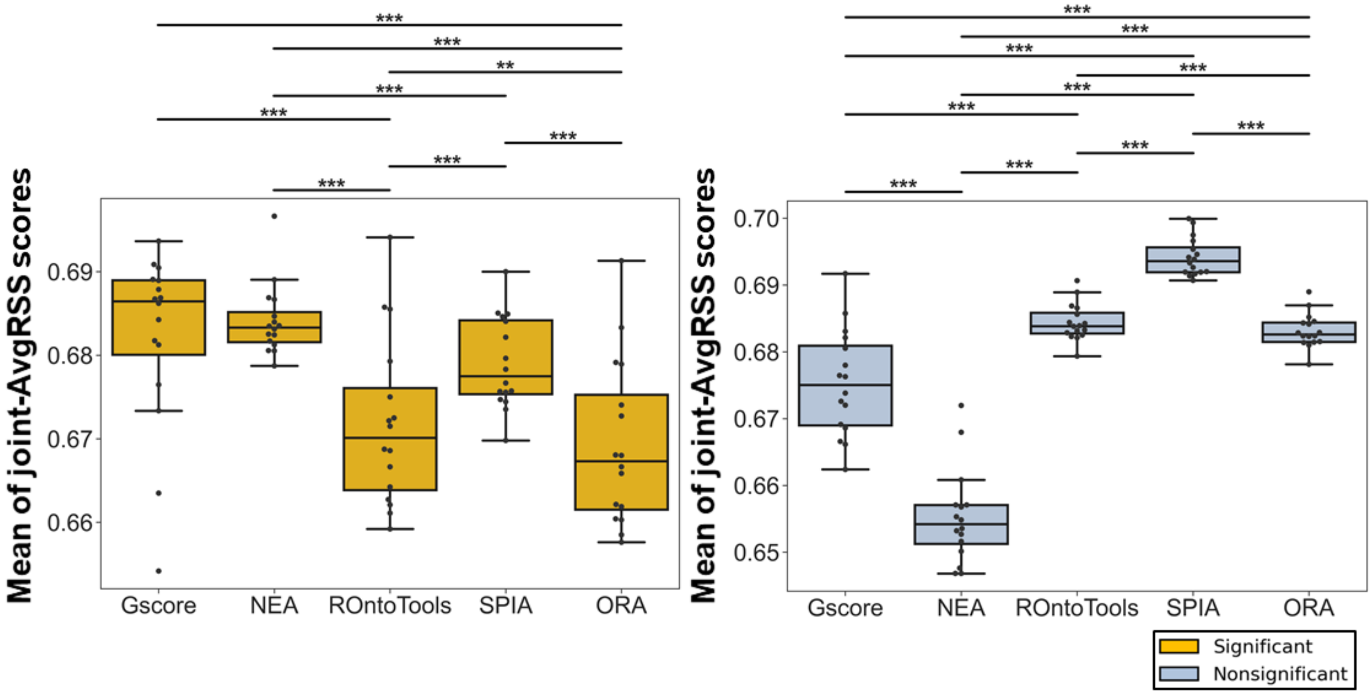

Supplementary Figure S5. Distributions of the joint average relative specificity similarity (AvgRSS) of GO biological process (BP) and cellular component (CC) terms for the significant (left) and nonsignificant (right) associations identified by the five methods using TCGA RNA-seq datasets in 16 cancers. Note that this analysis was performed only for those methods that use the DEG list as the input. Based on the GO BP and CC terms, we computed joint average RSS scores of the significant and nonsignificant associations to assess the shared biological functions of all gene pairs between the DEG list and each of the 69 pancancer pathways. For each gene pair, the RSS score for BP (or CC) was calculated first. Next, we measured the mean RSS scores for BP (or CC), called AvgRSS_{BP} (or AvgRSS_{CC}), across all gene pairs between the DEG list and pathway. Finally, the joint-AvgRSS score was evaluated based on the geometric mean, $\sqrt{\text{AvgRSS}_{BP} \times \text{AvgRSS}_{CC}}$. In every boxplot, each dot represents an individual data point, reflecting the mean of joint-AvgRSS scores across the significant (or nonsignificant) associations in each cancer type. *P* values of <0.01 and <0.001 (Wilcoxon signed-rank test) are indicated by a double asterisk and a triple asterisk, respectively.

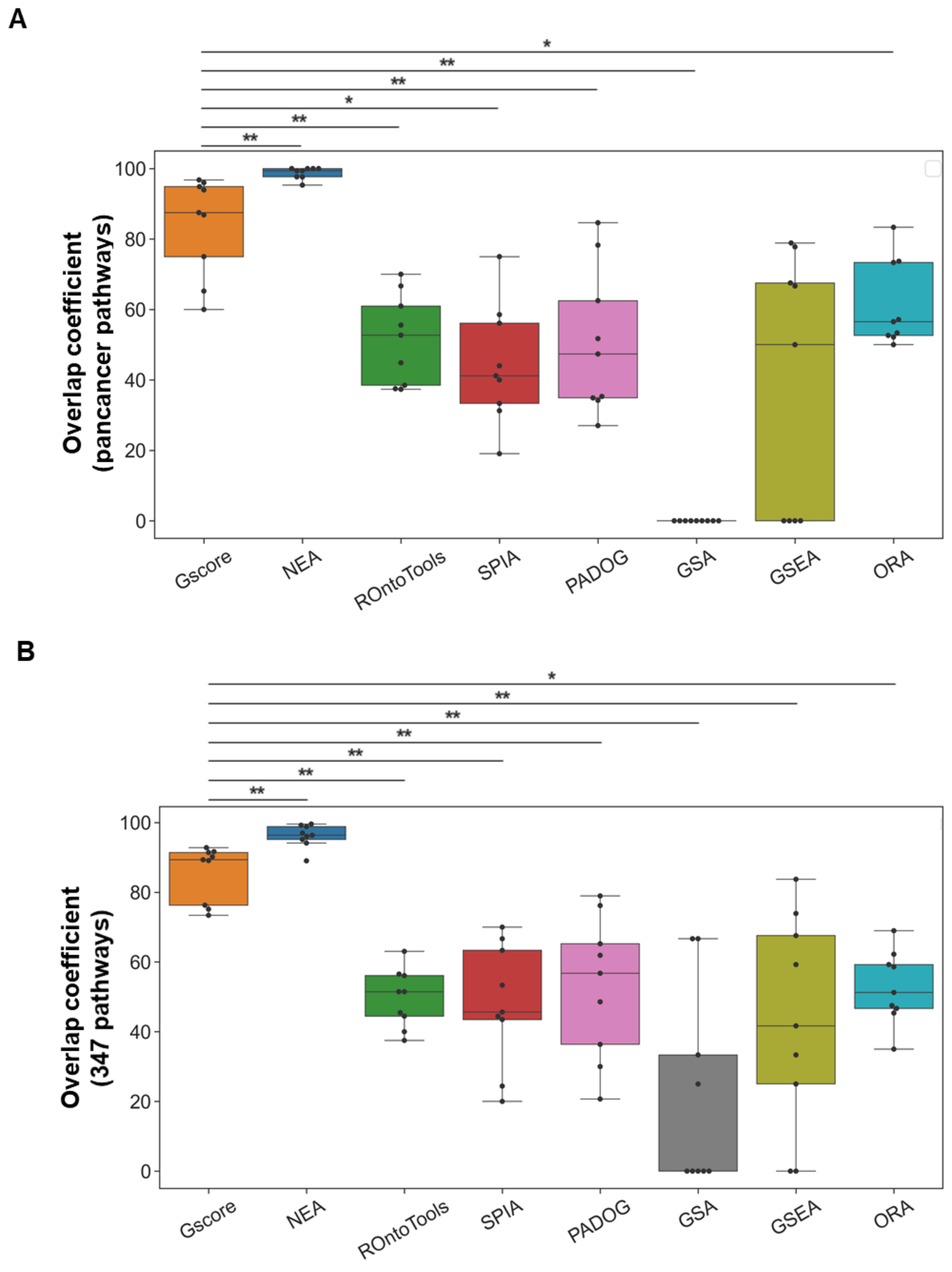

Supplementary Figure S6. Distribution of the overlap coefficient (OC) for significant associations with (A) 69 pancancer pathways and (B) 347 pathways identified by eight methods in the 9 TCGA RNA-seq and 45 GEO microarray datasets in nine shared cancer

types. In all boxplots, dots represent individual data points for nine cancer types. Note that there are nine cancer types represented in both the TCGA and GEO datasets. Since five microarray datasets are available for each cancer type, we evaluated the OC between all the nonredundant significant associations for these datasets and the significant associations for the corresponding TCGA RNA-seq dataset. The data for Gscore and the other methods were statistically analyzed using the Wilcoxon signed-rank test for paired data. *P* values of <0.05, <0.01 and <0.001 (Wilcoxon signed-rank test) are indicated by a single asterisk, a double asterisk and a triple asterisk, respectively.

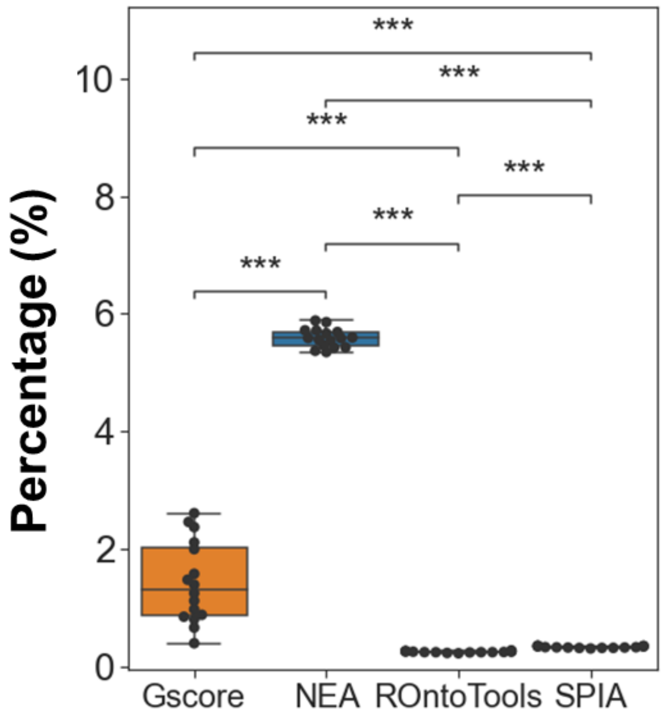

Supplementary Figure S7. Distributions of the percentages of significant associations among all possible pairs between 347 pathways and all individual DEGs derived from 16 RNA-seq datasets using four methods. Note that only the Gscore, NEA, ROntoTools, and SPIA methods, which can query an individual DEG, are available in this analysis. In all boxplots, dots represent individual data points for 16 cancer types. *P* values of <0.001 (Wilcoxon signed-rank test) are indicated by a triple asterisk.

A

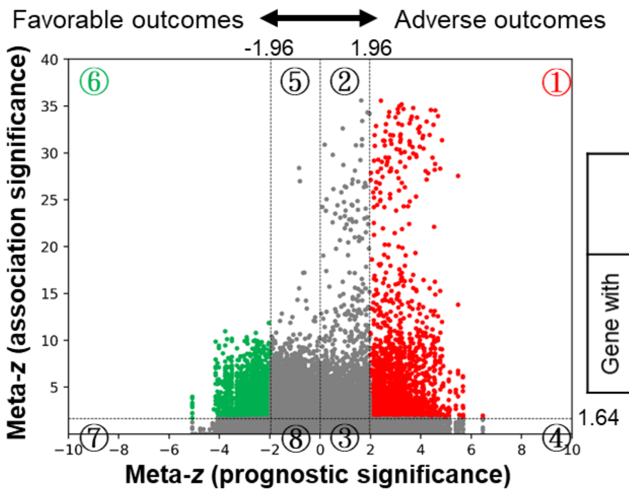

Numbers of associations		Meta-z (association significance) ≥1.64	Meta-z (association significance) <1.64
Gene with meta-z (prognostic significance)	≥ 1.96 or ≤ -1.96	① + ⑥	④ + ⑦
	>-1.96 and <1.96	② + ⑤	③ + ⑧

B

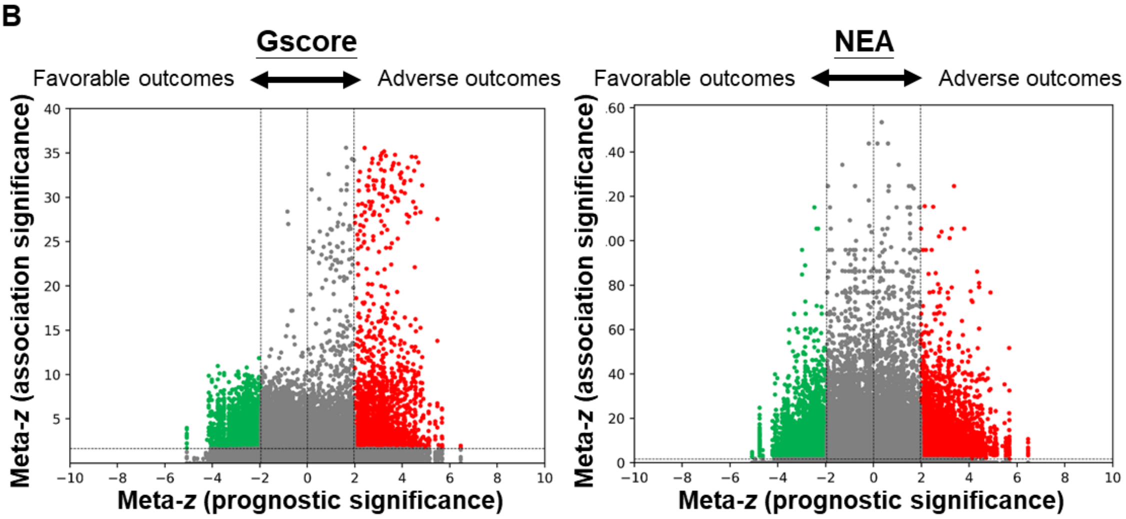

Gscore

Numbers of associations		Meta-z (association significance) ≥1.64	Meta-z (association significance) <1.64
Gene with meta-z (prognostic significance)	≥ 1.96 or ≤ -1.96	**3772**	**196052**
	>-1.96 and <1.96	**7980**	**770685**

p = 8×10^{-219} (Chi-square test)
Odds ratio = 1.858

NEA

Numbers of associations		Meta-z (association significance) ≥1.64	Meta-z (association significance) <1.64
Gene with meta-z (prognostic significance)	≥ 1.96 or ≤ -1.96	**12642**	**187182**
	>-1.96 and <1.96	**42350**	**736315**

p = 3×10^{-53} (Chi-square test)
Odds ratio = 1.174

Supplementary Figure S8. Statistical analysis of the proportion of adverse and favorable prognostic genes among the genes involved in the significant associations with the pancancer pathways across 16 cancer types. (A) Schematic diagram of the construction of the contingency table for examining the relationship between prognostic significance and association significance. **(B)** Volcano plot of the meta-*z* scores of adverse and favorable prognostic genes (*x*-axis) versus the meta-*z* scores of the FDR *q* values for associations between DEGs and pancancer pathways (*y*-axis) across 16 cancer types identified by Gscore (left) and NEA (right). The adverse and favorable prognostic genes (|meta-z| ≥ 1.96; nominal two-sided $p \leq 0.05$) involved in significant

associations across 16 cancer types (meta-z ≥1.64 or nominal one-sided $p \leq 0.05$) are represented by red and green dots, respectively. The gray dots represent nonsignificant associations (meta-z < 1.64) and nonsignificant prognostic genes (|meta-z| < 1.96). Chi-square tests and odds ratios were used to analyze the data in 2 × 2 contingency tables.

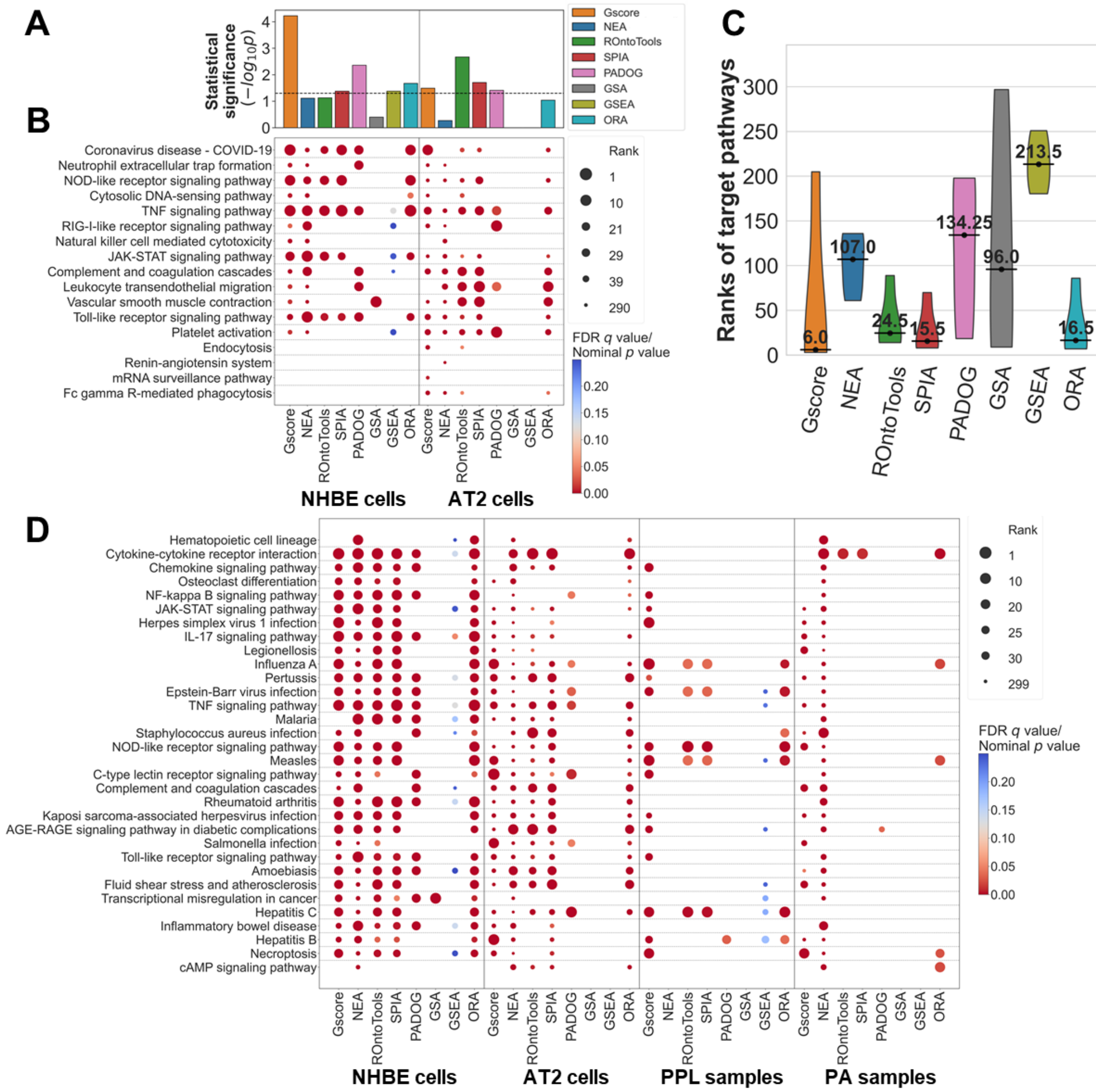

Supplementary Figure S9. Detections of associations between certain pathways and the DEG lists derived from four RNA-seq datasets relevant to COVID-19 infection using eight methods. **(A)** Bar chart (top panel) showing the statistical analysis of whether the proportion of COVID-19-related pathways among the significantly impacted pathways was higher than that among the nonsignificantly impacted pathways for two different DEG lists. The NHBE cell dataset contains three mock-treated and three SARS-CoV-2-infected primary NHBE cells. The AT2 cell dataset includes five mock-transfected and five SARS-CoV-2-infected organoids generated from primary lung AT2

cells. The PPL sample dataset consists of 100 plasma and leukocyte samples from hospitalized patients with COVID-19 and 26 samples from patients without COVID-19. The PA sample dataset comprises five autopsy lung samples from five control patients and 29 autopsy samples from eight patients with high SARS-CoV-2 loads. The dashed line indicates the statistical significance threshold of p value <0.05 ($-\log_{10} p > 1.301$, Fisher's exact test). **(B)** Dot plot showing the significant associations with COVID-19-related pathways in four RNA-seq datasets. The dot size is proportional to the rank of each COVID-19-related pathway identified by the corresponding method. The color bar indicates the nominal *p* value for the PADOG method and FDR *q* values for the other methods. **(C)** Violin plots showing the ranks of target pathways as determined by the eight methods using these four RNA-seq datasets; the value represents the median of ranks in each plot. Here, the pathway of 'coronavirus disease - COVID-19' (hsa05171) is considered the target pathway. **(D)** Dot plot showing the significant associations with 32 impacted pathways relevant to COVID-19 suggested by Draghici *et al.* (in Figures S6 and S7) (8) in four RNA-seq datasets. The dot size is proportional to the rank of each COVID-19-related pathway identified by the corresponding method. The color bar indicates the nominal *p* value for the PADOG method and FDR *q* values for the other methods.

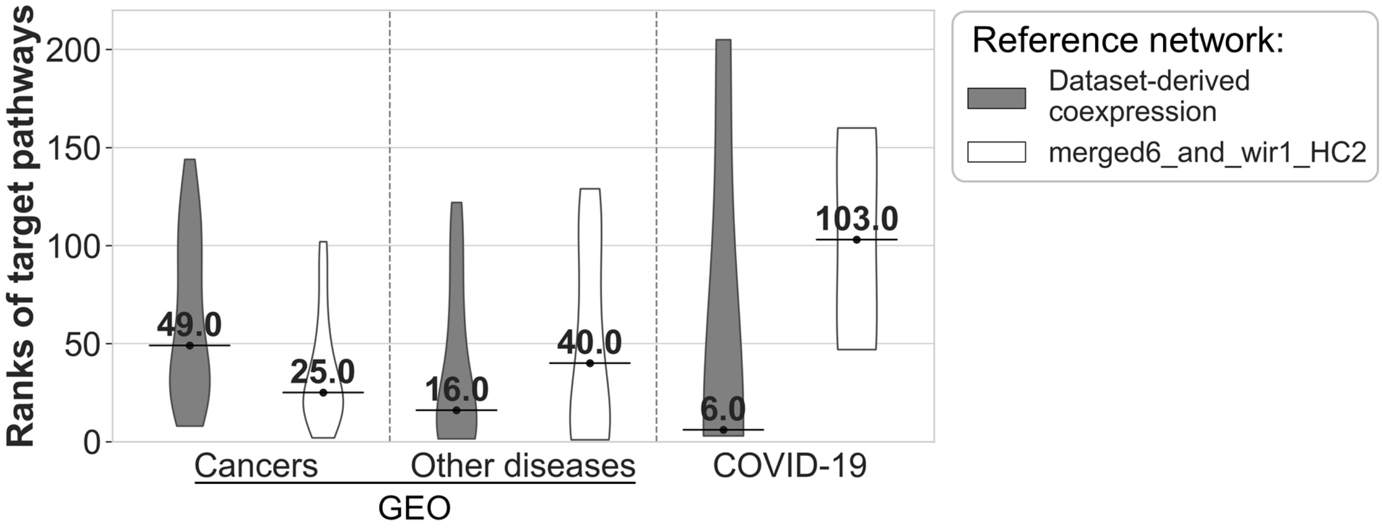

Supplementary Figure S10. Comparison of the ability to identify target pathways using Gscore based on the dataset-derived coexpression network (gray) or the merged6_and_wir1_HC2 network (white). Violin plots showing the ranks of target pathways identified by Gscore based on two different reference networks using 50 microarray datasets in 10 cancer types (left; details in Supplementary Table S1), 25 microarray datasets related to 5 other diseases (middle; e.g., Alzheimer disease, Parkinson disease, dilated cardiomyopathy, Huntington disease, type II diabetes mellitus), and 4 RNA-seq datasets relevant to COVID-19 infection (right; details in Supplementary Table S3). The aggregate network, merged6_ and_wir1 _HC2, was constructed by merging the higher confidence network 'FClim_HC2' with the curated links available from several databases (e.g., CORUM (9), Phosphosite (10), KEGG (11), and MSigDB (12)) and the reverse-engineered network 'wir1'. The FClim_HC2 network was assembled from the FunCoup (13) database. Notably, the wir1 network was derived from the TCGA glioblastoma expression, methylation, and mutation datasets and has been suggested to be beneficial for cancer data analysis (14). Among the collected datasets, those relevant to diseases that already have a

corresponding KEGG pathway (i.e., target pathway) were used. In each plot, the value represents the median of ranks across multiple datasets.

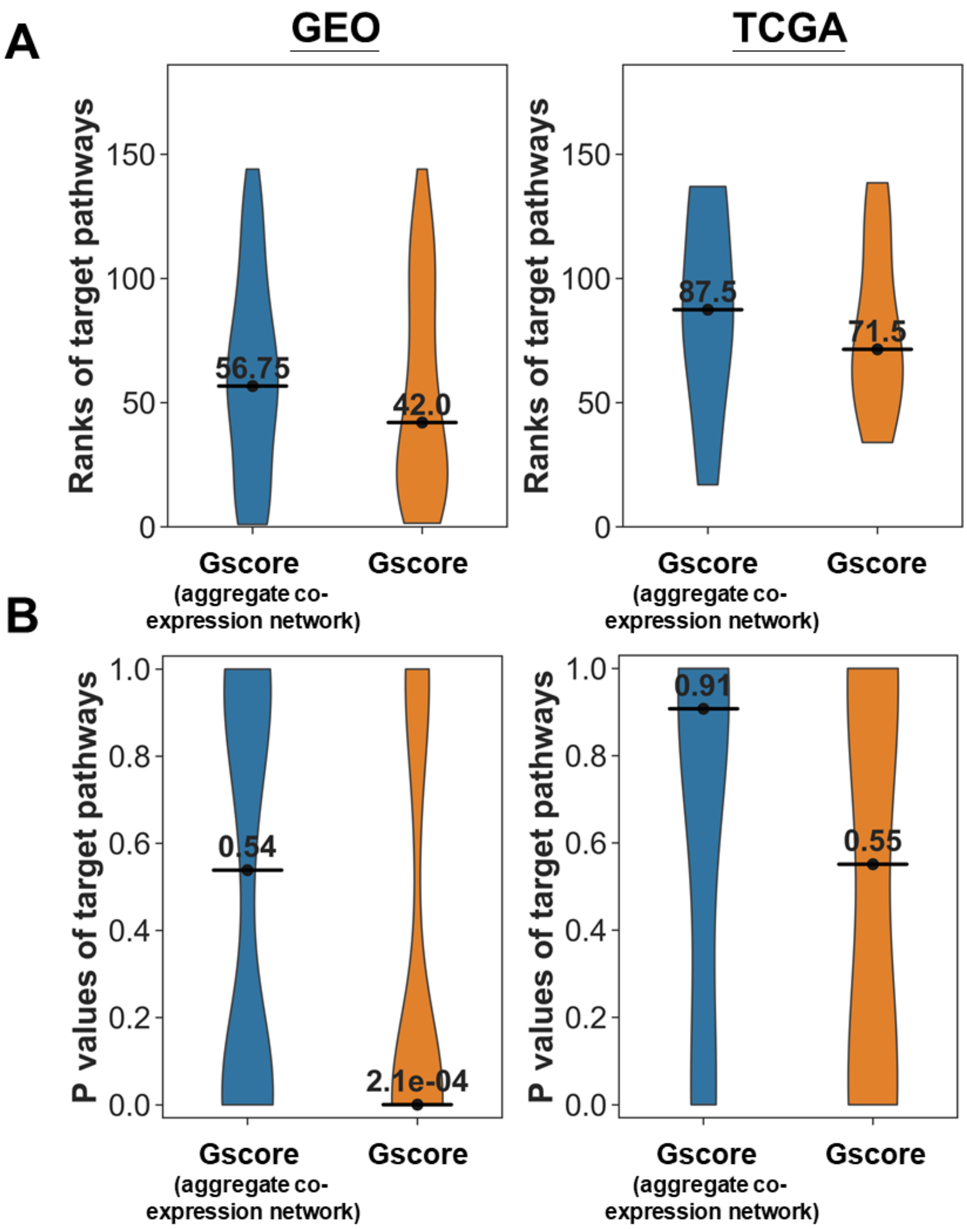

Supplementary Figure S11. Comparison of the ability to identify target pathways using Gscore based on the dataset-derived coexpression network (orange) or the aggregate coexpression network (blue). Violin plots showing **(A)** the ranks and **(B)** the *p* values of target pathways identified by Gscore based on two different coexpression networks using 75 GEO microarray datasets (left) and 10 TCGA RNA-seq datasets (right). The aggregate coexpression network (ver. Hsa-u.c4-0) derived from microarray and RNA-seq data of 245,698 samples was downloaded from the COXPRESdb (ver 8.1) database (15) (https://coxpresdb.jp/download/). Among the collected datasets, those relevant to diseases that already have a corresponding KEGG pathway (i.e., target pathway) were used. In each plot, the value represents the median of ranks (top) or p values (bottom) across the 75 GEO (or 10 TCGA) datasets.

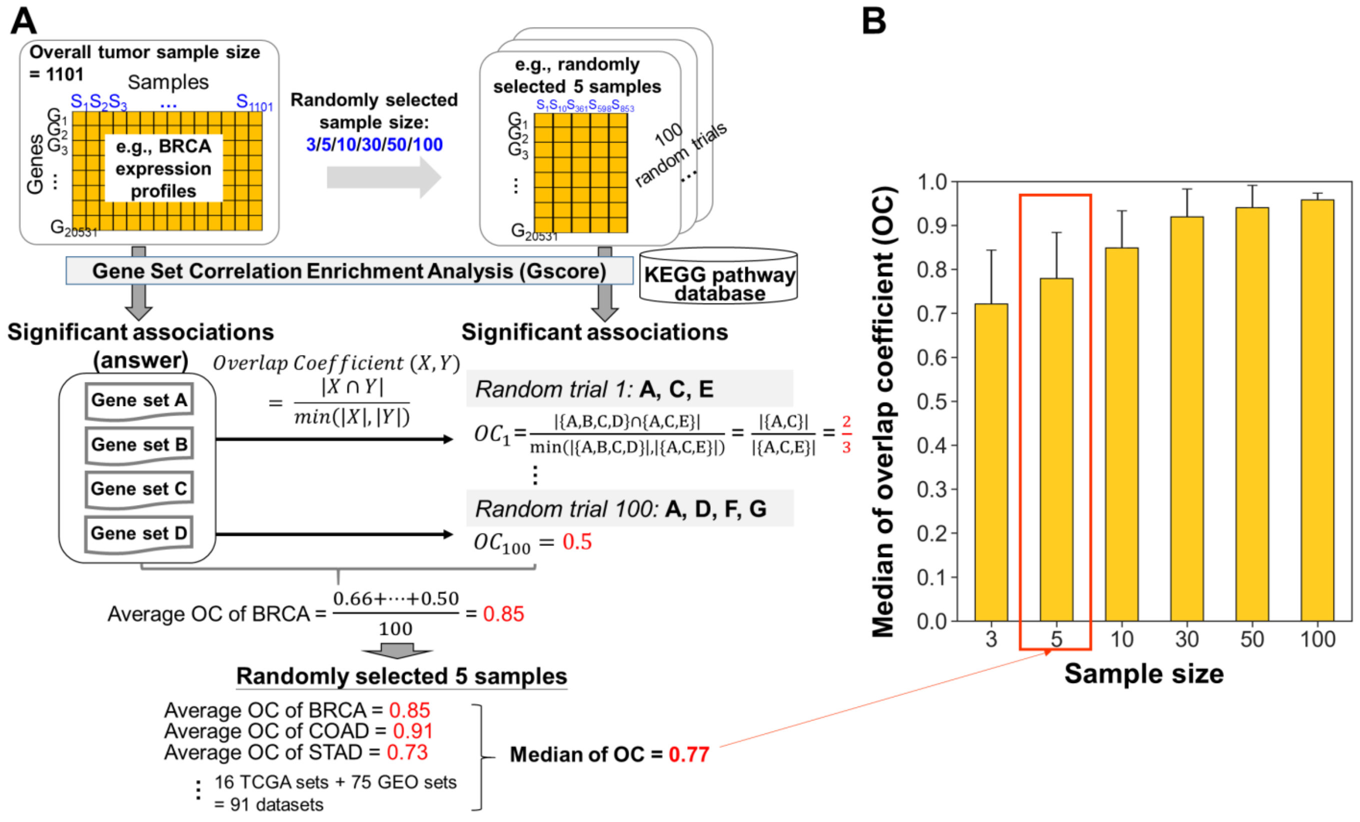

Supplementary Figure S12. Analysis of overlap coefficients (OCs) for significant associations detected by Gscore using the gene expression profiles across all samples and across randomly selected samples of different sizes. (A) Schematic diagram depicting the calculation of OC values between significant associations based on the gene expression profiles of overall samples and of randomly selected samples of different sizes (i.e., 3, 5, 10, 30, 50, and 100). Here, we used TCGA BRCA expression data as an example. For each of the 100 trials, we randomly selected five tumor samples from a total of 1101 BRCA samples and identified the DEGs between these five tumor samples (or all tumor samples) and all corresponding normal samples. The Gscore method was used to detect significant associations between these two DEG lists and 347 KEGG pathways. To examine the influence of the gene expression sample size for the Gscore method, we measured the OC values between significant associations identified using all tumor samples and those identified using selected samples in 100 random trials. **(B)** The median distribution of average OC values between significant associations detected using the reference set (i.e., all samples) and the random trial sets based on 16 TCGA RNA-seq datasets and 75 GEO microarray datasets. Error bars indicate standard deviation. The medians of the overlap coefficients (mOC) computed by using 91 datasets with corresponding random trials suggested that using at least five tumor samples (i.e.,

case sample; mOC ≥ 0.78) supported significantly greater performance than using only three samples (mOC =0.72; *p* value ≤ 9.7×10^{-5}, Mann–Whitney U test).

Supplementary Tables

Supplementary Table S1. Expression datasets of microarray in 15 diseases assembled from the Gene Expression Omnibus (GEO) database used in this study.

Disease	Dataset (GEO ID)	No. of control samples	No. of case samples	No. of DEGs[a]	Platform	References
Alzheimer's disease	GSE1297	9	7	2449	HG-U133A	Blalock, et al. (16)
	GSE5281EC	13	10	8217	HG-U133 Plus 2.0	Liang, et al. (17)<br>Liang, et al. (18)
	GSE5281HIP	13	10	7807	HG-U133 Plus 2.0	Redhead, et al. (19)
	GSE5281VCX	12	19	7031	HG-U133 Plus 2.0	Liang, et al. (20)
	GSE16759	4	4	922	HG-U133 Plus 2.0	Nunez-Iglesias, et al. (21)
Renal cell carcinoma	GSE14762	12	9	9162	HG-U133 Plus 2.0	Wang, et al (22).
	GSE781	5	12	3144	HG-U133A	Lenburg, et al. (23)
	GSE48352	8	24	6508	HG-U133 Plus 2.0	Ooi, et al. (unpublished)
	GSE6344	10	10	7267	HG-U133A	Gumz, et al. (24)<br>Tun, et al. (25)
	GSE6357	12	6	242	HG-U133A	Gigante, et al. (26)
Acute Myeloid Leukemia	GSE9476	37	26	4386	HG-U133A	Stirewalt, et al. (27)
	GSE14924_CD4	10	10	9462	HG-U133 Plus 2.0	Le Dieu, et al. (28)
	GSE14924_CD8	11	10	8030	HG-U133 Plus 2.0	Le Dieu, et al. (28)
	GSE68172	5	72	8446	HG-U133 Plus 2.0	Schneider, et al. (unpublished)
	GSE92778	6	6	2644	HuGene-10st	Boyd, et al. (29)
Chronic Myeloid Leukemia	GSE1418	6	8	424	HG-Focus	Neumann, et al. (30)
	GSE24739	8	16	4485	HG-U133 Plus 2.0	Affer, et al. (31)
	GSE24739_G0	4	8	4074	HG-U133 Plus 2.0	Abraham, et al. (32)
	GSE24739_G1	4	8	2275	HG-U133 Plus 2.0	
	GSE33075	9	18	4343	HG-U133 Plus 2.0	Benito, et al. (33)

Colorectal cancer	GSE4107	10	12	6889	HG-U133 Plus 2.0	Yi, et al. (34)
	GSE9348	12	70	12113	HG-U133 Plus 2.0	Hong, et al. (35)
	GSE23878	19	19	8550	HG-U133 Plus 2.0	Uddin, et al. (36)
	GSE4183	8	15	6240	HG-U133 Plus 2.0	Gyorffy, et al. (37)<br>Galamb, et al. (38)<br>Galamb, et al. (39)
	GSE8671	32	32	11594	HG-U133 Plus 2.0	Sabates-Bellver, et al. (40)
Dilated cardiomyopathy	GSE3585	5	7	1681	HG-U133A	Barth, et al. (41)
	GSE21610	8	42	5690	HG-U133 Plus 2.0	Schwientek, et al. (42)
	GSE29819	12	14	6618	HG-U133 Plus 2.0	Gaertner, et al. (43)
	GSE33970	18	5	692	HG-U133 Plus 2.0	Hollander, et al. (44)
	GSE79962	11	9	3781	HuGene-10st	Matkovich, et al. (45)
Endometrial cancer	GSE17025	12	91	6462	HG-U133 Plus 2.0	Day, et al. (46)<br>Day, et al. (47)
	GSE36389	7	13	822	HG-U133A	Orchel, et al. (unpublished)
	GSE63678	5	7	3122	HG-U133A	Pappa, et al. (48)
	GSE7305	10	10	10396	HG-U133 Plus 2.0	Hever, et al. (49)
	GSE7803	10	31	4976	HG-U133A	Zhai, et al. (50)
Glioma	GSE19728	4	17	7549	HG-U133 Plus 2.0	Liu, et al. (51)
	GSE21354	4	13	8941	HG-U133 Plus 2.0	Liu, et al. (51)
	GSE4290	23	157	13750	HG-U133 Plus 2.0	Bruna, et al. (52)
	GSE44971	9	49	15129	HG-U133 Plus 2.0	Lambert, et al. (53)
	GSE50161	13	95	11703	HG-U133 Plus 2.0	Griesinger, et al. (54)
Huntington's disease	GSE8762	10	12	586	HG-U133 Plus 2.0	Runne, et al. (55)
	GSE24250	6	8	362	HG-U133A	Hu, et al. (56)
	GSE37517	5	8	4021	HuGene-10st	HD iPSC Consortium. (57)

	GSE45516	3	6	8248	HG-U133 Plus 2.0	Marchina, et al. (58)
	GSE73655	7	13	1503	HuGene-10st	McCourt, et al. (59)
Non-small cell lung cancer	GSE18842	44	44	13174	HG-U133 Plus 2.0	Sanchez-Palencia, et al. (60)
	GSE19188	62	91	16382	HG-U133 Plus 2.0	Hou, et al. (61)
	GSE19804	60	60	12531	HG-U133 Plus 2.0	Lu, et al. (62)<br>Lu, et al. (63)
	GSE50627	6	9	8282	HuGene-10st	Zakaria, et al. (64)
	GSE6044	5	31	2015	HG-Focus	Rohrbeck, et al. (65)
Pancreatic cancer	GSE15471	35	35	16220	HG-U133 Plus 2.0	Badea, et al. (66)<br>Idichi, et al. (67)
	GSE16515	15	15	7568	HG-U133 Plus 2.0	Pei, et al. (68)<br>Li, et al. (69)<br>Ellsworth, et al. (70)
	GSE18670	6	18	8460	HG-U133 Plus 2.0	Sergeant, et al. (71)
	GSE28735	45	45	10545	HuGene-10st	Zhang, et al. (72)<br>Zhang, et al. (73)
	GSE32676	7	25	4823	HG-U133 Plus 2.0	Donahue, et al. (74)<br>Toste, et al. (75)
Parkinson disease	GSE20153	8	8	700	HG-U133 Plus 2.0	Zheng, et al. (76)
	GSE20291	20	15	681	HG-U133 Plus 2.0	Zhang, et al. (77)<br>Zheng, et al. (76)
	GSE19587	10	12	2326	HG-U133A 2	Lewandowski, et al. (78)
	GSE20164	5	6	643	HG-U133A	Zheng, et al. (76)
	GSE7621	9	16	2720	HG-U133A	Lesnick, et al. (79)
Prostate cancer	GSE6956AA	7	33	6449	HG-U133A 2	Wallace, et al. (80)
	GSE6956C	11	36	5980	HG-U133A 2	Wallace, et al. (80)
	GSE104749	4	4	3771	HG-U133 Plus 2.0	Shan, et al. (81)
	GSE26910	6	6	2082	HG-U133 Plus 2.0	Planche, et al. (82)
	GSE55945	7	12	4561	HG-U133 Plus 2.0	Arredouani, et al. (83)
Thyroid cancer	GSE3467	9	9	7343	HG-U133 Plus 2.0	He, et al. (84)
	GSE3678	7	7	2917	HG-U133 Plus 2.0	Reyes, et al. (unpublished)

	GSE58545	18	27	8043	HG-U133A	Rusinek, et al. (85)
	GSE58689	18	27	8043	HG-U133A	Rusinek, et al. (85)
	GSE85457	3	4	3988	HG-U133 Plus 2.0	Weinberger, et al. (unpublished)
Type II diabetes mellitusS	GSE19420	12	12	943	HG-U133 Plus 2.0	Van Tienen, et al. (86)
	GSE21340	15	5	918	HGU95Av2	Patti, et al. (87)
	GSE26887	5	7	3388	HuGene-10st	Greco, et al. (88)
	GSE38642	54	9	2344	HuGene-10st	Taneera, et al. (89)<br>Taneera, et al. (90)<br>Kanatsuna, et al. (91)<br>Hänzelmann, et al. (92)
	GSE39825	6	4	903	HGU95Av2	Knebel, et al. (93)

[a] For each microarray dataset, we followed the criteria from the previous work (94) to used p values ≤0.05 (two-sample t-test) for identifying DEGs.

Supplementary Table S2. Expression datasets of RNA-seq in 16 cancer types assembled from the Cancer Genome Atlas (TCGA) used in this study.

Name of cancer	Abbreviation	No. of normal samples	No. of tumor samples	No. of DEGs[a]
Bladder Urothelial Carcinoma	BLCA	19	408	4289
Breast invasive carcinoma	BRCA	113	1101	4248
Cervical squamous cell carcinoma and endocervical adenocarcinoma	CESC	3	305	4149
Cholangiocarcinoma	CHOL	8	36	6479
Colon adenocarcinoma	COAD	41	286	4819
Head and Neck squamous cell carcinoma	HNSC	44	522	3566
Kidney Chromophobe	KICH	25	65	5717
Kidney renal clear cell carcinoma	KIRC	72	534	4937
Liver hepatocellular carcinoma	LIHC	50	374	3687
Lung adenocarcinoma	LUAD	59	517	4430
Lung squamous cell carcinoma	LUSC	51	502	6365
Prostate adenocarcinoma	PRAD	52	497	2401
Rectum adenocarcinoma	READ	10	94	4664
Stomach adenocarcinoma	STAD	35	415	3472
Thyroid carcinoma	THCA	59	512	2614
Uterine Corpus Endometrial Carcinoma	UCEC	24	177	5476

[a] For each TCGA dataset, we followed the criteria from the previous works (4,7) to use $|\log_2(\text{fold change})| \geq 1$ and adjusted p values ≤ 0.05 (BH correction; limma) for identifying DEGs.

Supplementary Table S3. Expression datasets of RNA-seq relevant to COVID-19 assembled from the GEO database used in this study.

**Dataset (GEO ID)**	**Sample source**	**Abbreviation used in this study**	**No. of normal samples**	**No. of COVID-19 samples**	**No. of DEGs**	**References**
GSE150316	human lung	PA sample dataset	5	29	1106[a]	Desai, et al. (95)
GSE157103	human plasma and leukocyte	PPL sample dataset	26	100	291[b]	Overmyer, et al. (96)
GSE147507	NHBE_CoV2 (series 1)	NHBE cell dataset	3	3	128[a]	Hadjadj, et al. (97,98)
GSE160435	AT2 cells	AT2 cell dataset	5	5	3050[a]	Daamen, et al. (99)

[a] For GSE147507, GSE160435, and GSE150316 datasets, we followed the criteria from their corresponding works (95-99) to use p values <0.05 (Wald test; *DESeq2*) for identifying DEGs.
[b] For GSE157103 dataset, we determined the DEGs based on the same criteria [$|\log_2(\text{fold change})| \geq 1$ and adjusted p values ≤0.05 (BH correction; limma)] used in the TCGA datasets.

Supplementary Table S4. The target pathways for GEO microarray datasets used in this study.

Dataset (GEO ID)	Target pathway (KEGG ID)	Target pathway (pathway name)
GSE1297<br>GSE5281_EC<br>GSE5281_HIP<br>GSE5281_VCX<br>GSE16759	hsa05010	Alzheimer disease
GSE14762<br>GSE781<br>GSE48352<br>GSE6344<br>GSE6357	hsa05211	Renal cell carcinoma
GSE9476<br>GSE14924_CD4<br>GSE14924_CD8<br>GSE68172<br>GSE92778	hsa05221	Acute myeloid leukemia
GSE1418<br>GSE24739<br>GSE24739_G0<br>GSE24739_G1<br>GSE33075	hsa05220	Chronic myeloid leukemia
GSE4107<br>GSE9348<br>GSE23878<br>GSE4183<br>GSE8671	hsa05210	Colorectal cancer
GSE3585<br>GSE21610<br>GSE29819<br>GSE33970<br>GSE79962	hsa05414	Dilated cardiomyopathy
GSE17025<br>GSE36389<br>GSE63678<br>GSE7305<br>GSE7803	hsa05213	Endometrial cancer
GSE19728<br>GSE21354	hsa05214	Glioma

GSE4290<br>GSE44971<br>GSE50161		
GSE8762<br>GSE24250<br>GSE37517<br>GSE45516<br>GSE73655	hsa05016	Huntington disease
GSE18842<br>GSE19188<br>GSE19804<br>GSE50627<br>GSE6044	hsa05223	Non-small cell lung cancer
GSE15471<br>GSE16515<br>GSE18670<br>GSE28735<br>GSE32676	hsa05212	Pancreatic cancer
GSE20153<br>GSE20291<br>GSE19587<br>GSE20164<br>GSE7621	hsa05012	Parkinson disease
GSE6956_AA<br>GSE6956_C<br>GSE104749<br>GSE26910<br>GSE55945	hsa05215	Prostate cancer
GSE3467<br>GSE3678<br>GSE58545<br>GSE58689<br>GSE85457	hsa05216	Thyroid cancer
GSE19420<br>GSE21340<br>GSE26887<br>GSE38642<br>GSE39825	hsa04930	Type II diabetes mellitus

Supplementary Table S5. The target pathways for TCGA RNA-seq datasets used in this study.

TCGA	Target pathway (KEGG ID)	Target pathway (pathway name)
BLCA	hsa05219	Bladder cancer
COAD	hsa05210	Colorectal cancer
KICH	hsa05211	Renal cell carcinoma
KIRC	hsa05211	Renal cell carcinoma
LUAD	hsa05223	Non-small cell lung cancer
LUSC	hsa05223	Non-small cell lung cancer
PRAD	hsa05215	Prostate cancer
READ	hsa05210	Colorectal cancer
THCA	hsa05216	Thyroid cancer
UCEC	hsa05213	Endometrial cancer

Supplementary Table S6. List of pancancer pathways (69 in total), including 11 pathways that belong to the "6.1 Cancer: overview" category and 58 nonredundant related pathways as defined in the KEGG database.

6.1 Cancer: overview		Related pathway		Resource
KEGG ID	Pathway name	KEGG ID	Pathway name	
hsa05200	Pathways in cancer	hsa03320	PPAR signaling pathway	https://www.genome.jp/entry/hsa05200
		hsa04010	MAPK signaling pathway	
		hsa04020	Calcium signaling pathway	
		hsa04024	cAMP signaling pathway	
		hsa04060	Cytokine-cytokine receptor interaction	
		hsa04066	HIF-1 signaling pathway	
		hsa04110	Cell cycle	
		hsa04115	p53 signaling pathway	
		hsa04150	mTOR signaling pathway	
		hsa04151	PI3K-Akt signaling pathway	
		hsa04210	Apoptosis	
		hsa04310	Wnt signaling pathway	
		hsa04330	Notch signaling pathway	
		hsa04340	Hedgehog signaling pathway	
		hsa04350	TGF-beta signaling pathway	
		hsa04370	VEGF signaling pathway	
		hsa04510	Focal adhesion	
		hsa04512	ECM-receptor interaction	
		hsa04520	Adherens junction	
		hsa04630	JAK-STAT signaling pathway	
		hsa04915	Estrogen signaling pathway	
hsa05202	Transcriptional misregulation in cancer	hsa04115	p53 signaling pathway	https://www.genome.jp/entry/hsa05202
		hsa05211	Renal cell carcinoma	
		hsa05215	Prostate cancer	
		hsa05216	Thyroid cancer	
		hsa05221	Acute myeloid leukemia	
hsa05206	MicroRNAs in cancer	hsa05210	Colorectal cancer	https://www.genome.jp/entry/hsa05206
		hsa05214	Glioma	
		hsa05215	Prostate cancer	
		hsa05219	Bladder cancer	
		hsa05222	Small cell lung cancer	
		hsa05223	Non-small cell lung cancer	
hsa05205	Proteoglycans in cancer	hsa04010	MAPK signaling pathway	
		hsa04020	Calcium signaling pathway	

		hsa04066	HIF-1 signaling pathway	https://www.genome.jp/entry/hsa05205
		hsa04150	mTOR signaling pathway	
		hsa04151	PI3K-Akt signaling pathway	
		hsa04210	Apoptosis	
		hsa04310	Wnt signaling pathway	
		hsa04340	Hedgehog signaling pathway	
		hsa04350	TGF-beta signaling pathway	
		hsa04370	VEGF signaling pathway	
		hsa04510	Focal adhesion	
		hsa04520	Adherens junction	
		hsa04810	Regulation of actin cytoskeleton	
hsa05204	Chemical carcinogenesis - DNA adducts	hsa00980	Metabolism of xenobiotics by cytochrome P450	https://www.genome.jp/entry/hsa05204
		hsa05210	Colorectal cancer	
		hsa05211	Renal cell carcinoma	
		hsa05215	Prostate cancer	
		hsa05219	Bladder cancer	
		hsa05221	Acute myeloid leukemia	
		hsa05222	Small cell lung cancer	
		hsa05223	Non-small cell lung cancer	
		hsa05224	Breast cancer	
		hsa05225	Hepatocellular carcinoma	
hsa05207	Chemical carcinogenesis - receptor activation	hsa00980	Metabolism of xenobiotics by cytochrome P450	https://www.genome.jp/entry/hsa05207
		hsa03320	PPAR signaling pathway	
		hsa04010	MAPK signaling pathway	
		hsa04024	cAMP signaling pathway	
		hsa04150	mTOR signaling pathway	
		hsa04151	PI3K-Akt signaling pathway	
		hsa04370	VEGF signaling pathway	
		hsa04915	Estrogen signaling pathway	
		hsa05204	Chemical carcinogenesis - DNA adducts	
		hsa05206	MicroRNAs in cancer	
		hsa05208	Chemical carcinogenesis - reactive oxygen species	
hsa05208	Chemical carcinogenesis - reactive oxygen species	hsa00190	Oxidative phosphorylation	https://www.genome.jp/entry/hsa05208
		hsa00980	Metabolism of xenobiotics by cytochrome P450	
		hsa04010	MAPK signaling pathway	

		hsa04064	NF-kappa B signaling pathway	
		hsa04066	HIF-1 signaling pathway	
		hsa04151	PI3K-Akt signaling pathway	
		hsa04370	VEGF signaling pathway	
		hsa05207	Chemical carcinogenesis - receptor activation	
		hsa05211	Renal cell carcinoma	
		hsa05215	Prostate cancer	
		hsa05219	Bladder cancer	
		hsa05221	Acute myeloid leukemia	
		hsa05222	Small cell lung cancer	
		hsa05223	Non-small cell lung cancer	
		hsa05224	Breast cancer	
		hsa05225	Hepatocellular carcinoma	
hsa05203	Viral carcinogenesis	hsa03022	Basal transcription factors	https://www.genome.jp/entry/hsa05203
		hsa03320	PPAR signaling pathway	
		hsa04010	MAPK signaling pathway	
		hsa04062	Chemokine signaling pathway	
		hsa04064	NF-kappa B signaling pathway	
		hsa04110	Cell cycle	
		hsa04115	p53 signaling pathway	
		hsa04142	Lysosome	
		hsa04151	PI3K-Akt signaling pathway	
		hsa04210	Apoptosis	
		hsa04630	JAK-STAT signaling pathway	
		hsa04662	B cell receptor signaling pathway	
		hsa04810	Regulation of actin cytoskeleton	
		hsa05160	Hepatitis C	
		hsa05161	Hepatitis B	
		hsa05165	Human papillomavirus infection	
		hsa05166	Human T-cell leukemia virus 1 infection	
		hsa05167	Kaposi sarcoma-associated herpesvirus infection	
		hsa05169	Epstein-Barr virus infection	
hsa05230	Central carbon metabolism in cancer	hsa00010	Glycolysis / Gluconeogenesis	https://www.genome.jp/entry/hsa05230
		hsa00020	Citrate cycle (TCA cycle)	
		hsa00030	entose phosphate pathway	
		hsa00190	Oxidative phosphorylation	
		hsa00250	Alanine, aspartate and glutamate metabolism	

		hsa00260	Glycine, serine and threonine metabolism	
		hsa00330	Arginine and proline metabolism	
		hsa01212	Fatty acid metabolism	
		hsa04010	MAPK signaling pathway	
		hsa04066	HIF-1 signaling pathway	
		hsa04150	mTOR signaling pathway	
		hsa04151	PI3K-Akt signaling pathway	
hsa05231	Choline metabolism in cancer	hsa00564	Glycerophospholipid metabolism	https://www.genome.jp/entry/hsa05231
		hsa04010	MAPK signaling pathway	
		hsa04150	mTOR signaling pathway	
		hsa04151	PI3K-Akt signaling pathway	
		hsa04810	Regulation of actin cytoskeleton	
hsa05235	PD-L1 expression and PD-1 checkpoint pathway in cancer	hsa04010	MAPK signaling pathway	https://www.genome.jp/entry/hsa05235
		hsa04020	Calcium signaling pathway	
		hsa04066	HIF-1 signaling pathway	
		hsa04151	PI3K-Akt signaling pathway	
		hsa04514	Cell adhesion molecules	
		hsa04620	Toll-like receptor signaling pathway	
		hsa04660	T cell receptor signaling pathway	

Supplementary Table S7. COVID-19-related pathways derived from the pathway of "Coronavirus disease - COVID-19" and its related pathways defined by the KEGG database.

COVID-19 pathway		Related pathway		Resource
KEGG ID	Name	KEGG ID	Name	
hsa05171	Coronavirus disease - COVID-19	hsa04670	Leukocyte transendothelial migration	https://www.genome.jp/entry/hsa05171
		hsa04668	TNF signaling pathway	
		hsa04666	Fc gamma R-mediated phagocytosis	
		hsa04650	Natural killer cell mediated cytotoxicity	
		hsa04630	JAK-STAT signaling pathway	
		hsa04623	Cytosolic DNA-sensing pathway	
		hsa04622	RIG-I-like receptor signaling pathway	
		hsa04621	NOD-like receptor signaling pathway	
		hsa04620	Toll-like receptor signaling pathway	
		hsa04614	Renin-angiotensin system	
		hsa04613	Neutrophil extracellular trap formation	
		hsa04611	Platelet activation	
		hsa04610	Complement and coagulation cascades	
		hsa04270	Vascular smooth muscle contraction	
		hsa04144	Endocytosis	
		hsa03015	mRNA surveillance pathway	

Supplementary Table S8. Numbers of prognostic genes in TCGA 16 cancers.

TCGA abbreviated name	No. of prognostic genes (Adverse)	No. of prognostic genes (Favorable)	No. of patients
BLCA	830	1042	403
BRCA	1223	2071	1090
CESC	813	1107	303
CHOL	273	206	36
COAD	481	280	281
HNSC	1167	1483	518
KICH	773	736	63
KIRC	5574	3423	529
LIHC	790	550	369
LUAD	1033	1359	495
LUSC	913	515	486
PRAD	495	290	496
READ	485	831	92
STAD	881	183	407
THCA	693	477	503
UCEC	548	334	175

Supplementary Table S9. Introduction of the other topology-based (TB) and non-topology-based (non-TB) methods, including their tools and websites, used in this study.

Name	Summary	Web services [a] (Latest update)	Tool[b] (Latest version)	Type	Recommended cutoff
NEA (100)	Network Enrichment Analysis (NEA) is a network enrichment analysis approach that expands upon the overlap statistic in GEA to include network connections between genes in the experimentally derived altered gene set (e.g., DEG list) and those in the functional collections. Note that the suggested combined network, merged6_and_wir1_HC2 (downloaded from https://research.scilifelab.se/andrey_alexeyenko/downloads/evinet/merged6_and_wir1_HC2) (14), was used as input when implementing the NEA method by using the NEArender package (v. 1.5).	**EviNet** (101) (2018/01): https://www.evinet.org/	**NEArender** (102) (v. 1.5): https://rdrr.io/cran/NEArender/	TB	FDR *q* value ≤0.05
ROntoTools (103,104)	R Onto-Tools suite (ROntoTools) comprises the pioneering impact analysis approach, Pathway-Express (PE), and several subsequent improved methods built upon its foundation. It considers both a statistically significant number of DEGs and biologically meaningful alterations within a specified pathway.	1. **CPA** (105) (2021/02): https://bioinformatics.cse.unr.edu/software/cpa/pathway-analysis/ 2. **iPathwayGuide** (106) (2021/01): https://advaitabio.com/ipathwayguide/	**ROntoTools** (v. 2.28.0): https://bioconductor.org/packages/release/bioc/html/ROntoTools.html	TB	FDR *q* value ≤0.05
SPIA (107)	Signaling Pathway Impact Analysis (SPIA) employs data from a group of differentially expressed genes, including their fold changes, along with pathway topology to	**Graphite** (108) (2012/01):	**SPIA** (v. 2.52.0):	TB	FDR *q* value ≤0.05

	evaluate the significance of pathways in the studied condition. Specifically, it integrates two evidence types: the over-representation of differentially expressed genes in a specific pathway and the abnormal perturbation observed in that pathway (i.e., propagating measured expression changes across the pathway topology).	https://graphiteweb.bio.unipd.it/analyze.html	https://bioconductor.org/packages/release/bioc/html/SPIA.html		
CePa (109,110)	Centrality-based Pathway Analysis (CePa) extends standard pathway analysis methods (i.e., GSA and ORA) by considering two aspects: (i) pathway node is considered as the basic unit instead of a single gene when analyzing networks to satisfy that genes often work together within functional complexes to maintain regular functions; (ii) multiple network centralities are applied simultaneously to estimate the importance of nodes. Note that the KEGG pathway data were already embedded in the CePa tool, but the version is unknown.	NA	**CePa (**v. 0.8.0): https://cran.r-project.org/web/packages/CePa/index.html	TB	CePaORA: FDR *q* value ≤0.05 CePaGSA: FDR *q* value ≤0.1
PADOG (111)	Pathway Analysis with Down-weighting of Overlapping Genes (PADOG) computes a gene set score by averaging the absolute values of weighted moderated gene t-scores. The gene weights are used to prioritize the genes that appear in a few gene sets rather than in multiple gene sets.	**CPA** (2021/02): https://bioinformatics.cse.unr.edu/software/cpa/pathway-analysis/	**PADOG** (v.1.42.0): https://www.bioconductor.org/packages/release/bioc/html/PADOG.html	non-TB[c]	Nominal *p* value <0.05
GSA (112)	The Gene Set Analysis (GSA) method extends the GSEA method by introducing row randomization, column permutation, and *restandardization* of the *maxmean* statistic to establish a unique null distribution for accurate estimation of false discovery rates.	**CPA** (2021/02): https://bioinformatics.cse.unr.edu/software/cpa/pathway-analysis/	**GSA** (v. 1.03.2): https://cran.r-project.org/web/packages/GSA/index.html	non-TB[c]	FDR *q* value ≤0.1

GSEA (113)	Gene Set Enrichment Analysis (GSEA) is a computational method that determines whether an a priori defined set of genes shows statistically significant, concordant differences between two biological states (e.g., phenotypes). GSEA takes into account the collective behavior of genes within pre-defined gene sets or pathways. It ranks all genes based on their differential expression between groups and then assesses whether genes within a gene set or pathway are enriched at the top or bottom of the ranked list.	1. **WebGestalt** (114) (2021/06): https://www.webgestalt.org/<br>2. **CPA** (2021/02): https://bioinformatics.cse.unr.edu/software/cpa/pathway-analysis/	1. **GSEA software** (v. 4.3.2): https://www.gsea-msigdb.org/gsea/index.jsp<br>2. **GSEA_R** (v. 1.2): https://github.com/GSEA-MSigDB/GSEA_R<br>3. **clusterProfiler** (115) (v. 4.0): https://github.com/YuLab-SMU/clusterProfiler<br>4. **WebGestaltR** (v. 0.4.6): https://cran.r-project.org/web/packages/WebGestaltR/index.html	non-TB[c]	FDR *q* value ≤0.25

ORA[d] (116-119)	Over Representation Analysis (ORA) is a commonly employed method in bioinformatics and genomics research to determine the enrichment of specific biological functions or processes within a given set of gene or protein set. This analysis involves comparing the frequency of genes or proteins belonging to a particular category or annotation in the input gene set against the expected frequency by chance. In this study, we estimated the statistical significance in the ORA method based on the hypergeometric distribution using hypergeom.sf function from the SciPy package (v. 1.9.1)	1. **WebGestalt** (2021/06): https://www.webgestalt.org/# https://metascape.org/gp/index.html#/main/step1 1. **DAVID** (120,121) (2023/06) https://david.ncifcrf.gov/home.jsp	1. **WebGestaltR** (v. 0.4.6): https://cran.r-project.org/web/packages/WebGestaltR/index.html 2. **GOstats** (119) (v. 2.66.0) https://bioconductor.org/packages/release/bioc/html/GOstats.html 3. **clusterProfiler** (v. 4.0): https://github.com/YuLab-SMU/clusterProfiler	non-TB[c]	FDR *q* value ≤0.05

[a] Note that not all the web services were listed here for each method. We list only the web services offered by the same developers as the original method or commonly used and currently available services.

[b] Note that not all the tools were listed here for each method. We list only the tools offered by the same developers as the original method or commonly used and currently available tools.

[c] Over-representation analysis (ORA) and functional class scoring (FCS) methods are the first and second generations in the non-TB category (94), respectively. For example, GSA, GSEA, and PADOG belong to FCS methods.

[d] Many ORA-based tools have been presented since 2002; here, we only cited the paper from the early proposed tools or typical and commonly used ones.

REFERENCES

1. Ashburner, M., Ball, C.A., Blake, J.A., Botstein, D., Butler, H., Cherry, J.M., Davis, A.P., Dolinski, K., Dwight, S.S., Eppig, J.T. *et al.* (2000) Gene ontology: tool for the unification of biology. The Gene Ontology Consortium. *Nat Genet*, **25**, 25-29.
2. Wu, X., Zhu, L., Guo, J., Zhang, D.Y. and Lin, K. (2006) Prediction of yeast protein-protein interaction network: insights from the Gene Ontology and annotations. *Nucleic Acids Res*, **34**, 2137-2150.
3. Lin, C.Y., Lin, Y.W., Yu, S.W., Lo, Y.S. and Yang, J.M. (2012) MoNetFamily: a web server to infer homologous modules and module-module interaction networks in vertebrates. *Nucleic Acids Res*, **40**, W263-270.
4. Lin, C.Y., Lee, C.H., Chuang, Y.H., Lee, J.Y., Chiu, Y.Y., Wu Lee, Y.H., Jong, Y.J., Hwang, J.K., Huang, S.H., Chen, L.C. *et al.* (2019) Membrane protein-regulated networks across human cancers. *Nat Commun*, **10**, 3131.
5. Chen, C.C., Lin, C.Y., Lo, Y.S. and Yang, J.M. (2009) PPISearch: a web server for searching homologous protein-protein interactions across multiple species. *Nucleic Acids Res*, **37**, W369-375.
6. Clark, N.R., Hu, K.S., Feldmann, A.S., Kou, Y., Chen, E.Y., Duan, Q. and Ma'ayan, A. (2014) The characteristic direction: a geometrical approach to identify differentially expressed genes. *BMC Bioinformatics*, **15**, 79.
7. Chen, H.H., Hsueh, C.W., Lee, C.H., Hao, T.Y., Tu, T.Y., Chang, L.Y., Lee, J.C. and Lin, C.Y. (2023) SWEET: a single-sample network inference method for deciphering individual features in disease. *Brief Bioinform*, **24**.
8. Draghici, S., Nguyen, T.M., Sonna, L.A., Ziraldo, C., Vanciu, R., Fadel, R., Morrison, A., Kenney, R.M., Alangaden, G., Ramesh, M. *et al.* (2021) COVID-19: disease pathways and gene expression changes predict methylprednisolone can improve outcome in severe cases. *Bioinformatics*, **37**, 2691-2698.
9. Giurgiu, M., Reinhard, J., Brauner, B., Dunger-Kaltenbach, I., Fobo, G., Frishman, G., Montrone, C. and Ruepp, A. (2019) CORUM: the comprehensive resource of mammalian protein complexes-2019. *Nucleic Acids Res*, **47**, D559-D563.
10. Hornbeck, P.V., Kornhauser, J.M., Tkachev, S., Zhang, B., Skrzypek, E., Murray, B., Latham, V. and Sullivan, M. (2012) PhosphoSitePlus: a comprehensive resource for investigating the structure and function of experimentally determined post-translational modifications in man and mouse. *Nucleic Acids Research*, **40**, D261-D270.
11. Kanehisa, M. and Goto, S. (2000) KEGG: kyoto encyclopedia of genes and genomes. *Nucleic Acids Res*, **28**, 27-30.
12. Liberzon, A., Birger, C., Thorvaldsdottir, H., Ghandi, M., Mesirov, J.P. and Tamayo, P. (2015) The Molecular Signatures Database (MSigDB) hallmark gene set collection. *Cell Syst*, **1**, 417-425.
13. Alexeyenko, A. and Sonnhammer, E.L. (2009) Global networks of functional coupling in eukaryotes from comprehensive data integration. *Genome Res*, **19**, 1107-1116.

14. Merid, S.K., Goranskaya, D. and Alexeyenko, A. (2014) Distinguishing between driver and passenger mutations in individual cancer genomes by network enrichment analysis. *BMC Bioinformatics*, **15**, 308.
15. Obayashi, T., Kodate, S., Hibara, H., Kagaya, Y. and Kinoshita, K. (2023) COXPRESdb v8: an animal gene coexpression database navigating from a global view to detailed investigations. *Nucleic Acids Res*, **51**, D80-D87.
16. Blalock, E.M., Geddes, J.W., Chen, K.C., Porter, N.M., Markesbery, W.R. and Landfield, P.W. (2004) Incipient Alzheimer's disease: microarray correlation analyses reveal major transcriptional and tumor suppressor responses. *Proc Natl Acad Sci U S A*, **101**, 2173-2178.
17. Liang, W.S., Dunckley, T., Beach, T.G., Grover, A., Mastroeni, D., Walker, D.G., Caselli, R.J., Kukull, W.A., McKeel, D., Morris, J.C. *et al.* (2007) Gene expression profiles in anatomically and functionally distinct regions of the normal aged human brain. *Physiol Genomics*, **28**, 311-322.
18. Liang, W.S., Reiman, E.M., Valla, J., Dunckley, T., Beach, T.G., Grover, A., Niedzielko, T.L., Schneider, L.E., Mastroeni, D., Caselli, R. *et al.* (2008) Alzheimer's disease is associated with reduced expression of energy metabolism genes in posterior cingulate neurons. *Proc Natl Acad Sci U S A*, **105**, 4441-4446.
19. Readhead, B., Haure-Mirande, J.V., Funk, C.C., Richards, M.A., Shannon, P., Haroutunian, V., Sano, M., Liang, W.S., Beckmann, N.D., Price, N.D. *et al.* (2018) Multiscale Analysis of Independent Alzheimer's Cohorts Finds Disruption of Molecular, Genetic, and Clinical Networks by Human Herpesvirus. *Neuron*, **99**, 64-82 e67.
20. Liang, W.S., Dunckley, T., Beach, T.G., Grover, A., Mastroeni, D., Ramsey, K., Caselli, R.J., Kukull, W.A., McKeel, D., Morris, J.C. *et al.* (2008) Altered neuronal gene expression in brain regions differentially affected by Alzheimer's disease: a reference data set. *Physiol Genomics*, **33**, 240-256.
21. Nunez-Iglesias, J., Liu, C.C., Morgan, T.E., Finch, C.E. and Zhou, X.J. (2010) Joint genome-wide profiling of miRNA and mRNA expression in Alzheimer's disease cortex reveals altered miRNA regulation. *PLoS One*, **5**, e8898.
22. Wang, Y., Roche, O., Yan, M.S., Finak, G., Evans, A.J., Metcalf, J.L., Hast, B.E., Hanna, S.C., Wondergem, B., Furge, K.A. *et al.* (2009) Regulation of endocytosis via the oxygen-sensing pathway. *Nat Med*, **15**, 319-324.
23. Lenburg, M.E., Liou, L.S., Gerry, N.P., Frampton, G.M., Cohen, H.T. and Christman, M.F. (2003) Previously unidentified changes in renal cell carcinoma gene expression identified by parametric analysis of microarray data. *BMC Cancer*, **3**, 31.
24. Gumz, M.L., Zou, H., Kreinest, P.A., Childs, A.C., Belmonte, L.S., LeGrand, S.N., Wu, K.J., Luxon, B.A., Sinha, M., Parker, A.S. *et al.* (2007) Secreted frizzled-related protein 1 loss contributes to tumor phenotype of clear cell renal cell carcinoma. *Clin Cancer Res*, **13**, 4740-4749.
25. Tun, H.W., Marlow, L.A., von Roemeling, C.A., Cooper, S.J., Kreinest, P., Wu, K., Luxon, B.A., Sinha, M., Anastasiadis, P.Z. and Copland, J.A. (2010) Pathway signature and cellular differentiation in clear cell renal cell carcinoma. *PLoS One*, **5**, e10696.

26. Gigante, M., Pontrelli, P., Herr, W., Gigante, M., D'Avenia, M., Zaza, G., Cavalcanti, E., Accetturo, M., Lucarelli, G., Carrieri, G. *et al.* (2016) miR-29b and miR-198 overexpression in CD8+ T cells of renal cell carcinoma patients down-modulates JAK3 and MCL-1 leading to immune dysfunction. *J Transl Med*, **14**, 84.
27. Stirewalt, D.L., Meshinchi, S., Kopecky, K.J., Fan, W., Pogosova-Agadjanyan, E.L., Engel, J.H., Cronk, M.R., Dorcy, K.S., McQuary, A.R., Hockenbery, D. *et al.* (2008) Identification of genes with abnormal expression changes in acute myeloid leukemia. *Genes Chromosomes Cancer*, **47**, 8-20.
28. Le Dieu, R., Taussig, D.C., Ramsay, A.G., Mitter, R., Miraki-Moud, F., Fatah, R., Lee, A.M., Lister, T.A. and Gribben, J.G. (2009) Peripheral blood T cells in acute myeloid leukemia (AML) patients at diagnosis have abnormal phenotype and genotype and form defective immune synapses with AML blasts. *Blood*, **114**, 3909-3916.
29. Boyd, A.L., Reid, J.C., Salci, K.R., Aslostovar, L., Benoit, Y.D., Shapovalova, Z., Nakanishi, M., Porras, D.P., Almakadi, M., Campbell, C.J.V. *et al.* (2017) Acute myeloid leukaemia disrupts endogenous myelo-erythropoiesis by compromising the adipocyte bone marrow niche. *Nat Cell Biol*, **19**, 1336-1347.
30. Neumann, F., Teutsch, N., Kliszewski, S., Bork, S., Steidl, U., Brors, B., Schimkus, N., Roes, N., Germing, U., Hildebrandt, B. *et al.* (2005) Gene expression profiling of Philadelphia chromosome (Ph)-negative CD34+ hematopoietic stem and progenitor cells of patients with Ph-positive CML in major molecular remission during therapy with imatinib. *Leukemia*, **19**, 458-460.
31. Affer, M., Dao, S., Liu, C., Olshen, A.B., Mo, Q., Viale, A., Lambek, C.L., Marr, T.G. and Clarkson, B.D. (2011) Gene Expression Differences between Enriched Normal and Chronic Myelogenous Leukemia Quiescent Stem/Progenitor Cells and Correlations with Biological Abnormalities. *J Oncol*, **2011**, 798592.
32. Abraham, S.A., Hopcroft, L.E., Carrick, E., Drotar, M.E., Dunn, K., Williamson, A.J., Korfi, K., Baquero, P., Park, L.E., Scott, M.T. *et al.* (2016) Dual targeting of p53 and c-MYC selectively eliminates leukaemic stem cells. *Nature*, **534**, 341-346.
33. Benito, R., Lumbreras, E., Abaigar, M., Gutierrez, N.C., Delgado, M., Robledo, C., Garcia, J.L., Rodriguez-Vicente, A.E., Canizo, M.C. and Rivas, J.M. (2012) Imatinib therapy of chronic myeloid leukemia restores the expression levels of key genes for DNA damage and cell-cycle progression. *Pharmacogenet Genomics*, **22**, 381-388.
34. Hong, Y., Ho, K.S., Eu, K.W. and Cheah, P.Y. (2007) A susceptibility gene set for early onset colorectal cancer that integrates diverse signaling pathways: implication for tumorigenesis. *Clin Cancer Res*, **13**, 1107-1114.
35. Hong, Y., Downey, T., Eu, K.W., Koh, P.K. and Cheah, P.Y. (2010) A 'metastasis-prone' signature for early-stage mismatch-repair proficient sporadic colorectal cancer patients and its implications for possible therapeutics. *Clin Exp Metastasis*, **27**, 83-90.
36. Uddin, S., Ahmed, M., Hussain, A., Abubaker, J., Al-Sanea, N., AbdulJabbar, A., Ashari, L.H., Alhomoud, S., Al-Dayel, F., Jehan, Z. *et al.* (2011) Genome-wide expression analysis of Middle Eastern colorectal cancer reveals FOXM1 as a novel target for cancer therapy. *Am J Pathol*, **178**, 537-547.

37. Gyorffy, B., Molnar, B., Lage, H., Szallasi, Z. and Eklund, A.C. (2009) Evaluation of microarray preprocessing algorithms based on concordance with RT-PCR in clinical samples. *PLoS One*, **4**, e5645.
38. Galamb, O., Gyorffy, B., Sipos, F., Spisak, S., Nemeth, A.M., Miheller, P., Tulassay, Z., Dinya, E. and Molnar, B. (2008) Inflammation, adenoma and cancer: objective classification of colon biopsy specimens with gene expression signature. *Dis Markers*, **25**, 1-16.
39. Galamb, O., Wichmann, B., Sipos, F., Spisak, S., Krenacs, T., Toth, K., Leiszter, K., Kalmar, A., Tulassay, Z. and Molnar, B. (2012) Dysplasia-carcinoma transition specific transcripts in colonic biopsy samples. *PLoS One*, **7**, e48547.
40. Sabates-Bellver, J., Van der Flier, L.G., de Palo, M., Cattaneo, E., Maake, C., Rehrauer, H., Laczko, E., Kurowski, M.A., Bujnicki, J.M., Menigatti, M. *et al.* (2007) Transcriptome profile of human colorectal adenomas. *Mol Cancer Res*, **5**, 1263-1275.
41. Barth, A.S., Kuner, R., Buness, A., Ruschhaupt, M., Merk, S., Zwermann, L., Kaab, S., Kreuzer, E., Steinbeck, G., Mansmann, U. *et al.* (2006) Identification of a common gene expression signature in dilated cardiomyopathy across independent microarray studies. *J Am Coll Cardiol*, **48**, 1610-1617.
42. Schwientek, P., Ellinghaus, P., Steppan, S., D'Urso, D., Seewald, M., Kassner, A., Cebulla, R., Schulte-Eistrup, S., Morshuis, M., Rofe, D. *et al.* (2010) Global gene expression analysis in nonfailing and failing myocardium pre- and postpulsatile and nonpulsatile ventricular assist device support. *Physiol Genomics*, **42**, 397-405.
43. Gaertner, A., Schwientek, P., Ellinghaus, P., Summer, H., Golz, S., Kassner, A., Schulz, U., Gummert, J. and Milting, H. (2012) Myocardial transcriptome analysis of human arrhythmogenic right ventricular cardiomyopathy. *Physiol Genomics*, **44**, 99-109.
44. Hollander, Z., Chen, V., Sidhu, K., Lin, D., Ng, R.T., Balshaw, R., Cohen-Freue, G.V., Ignaszewski, A., Imai, C., Kaan, A. *et al.* (2013) Predicting acute cardiac rejection from donor heart and pre-transplant recipient blood gene expression. *J Heart Lung Transplant*, **32**, 259-265.
45. Matkovich, S.J., Al Khiami, B., Efimov, I.R., Evans, S., Vader, J., Jain, A., Brownstein, B.H., Hotchkiss, R.S. and Mann, D.L. (2017) Widespread Down-Regulation of Cardiac Mitochondrial and Sarcomeric Genes in Patients With Sepsis. *Crit Care Med*, **45**, 407-414.
46. Day, R.S., McDade, K.K., Chandran, U.R., Lisovich, A., Conrads, T.P., Hood, B.L., Kolli, V.S., Kirchner, D., Litzi, T. and Maxwell, G.L. (2011) Identifier mapping performance for integrating transcriptomics and proteomics experimental results. *BMC Bioinformatics*, **12**, 213.
47. Day, R.S. and McDade, K.K. (2013) A decision theory paradigm for evaluating identifier mapping and filtering methods using data integration. *BMC Bioinformatics*, **14**, 223.
48. Pappa, K.I., Polyzos, A., Jacob-Hirsch, J., Amariglio, N., Vlachos, G.D., Loutradis, D. and Anagnou, N.P. (2015) Profiling of Discrete Gynecological Cancers Reveals Novel Transcriptional Modules and Common Features Shared by Other Cancer Types and Embryonic Stem Cells. *PLoS One*, **10**, e0142229.
49. Hever, A., Roth, R.B., Hevezi, P., Marin, M.E., Acosta, J.A., Acosta, H., Rojas, J., Herrera, R., Grigoriadis, D., White, E. *et al.* (2007) Human endometriosis is associated with plasma

cells and overexpression of B lymphocyte stimulator. *Proc Natl Acad Sci U S A*, **104**, 12451-12456.
50. Zhai, Y., Kuick, R., Nan, B., Ota, I., Weiss, S.J., Trimble, C.L., Fearon, E.R. and Cho, K.R. (2007) Gene expression analysis of preinvasive and invasive cervical squamous cell carcinomas identifies HOXC10 as a key mediator of invasion. *Cancer Res*, **67**, 10163-10172.
51. Liu, Z., Yao, Z., Li, C., Lu, Y. and Gao, C. (2011) Gene expression profiling in human high-grade astrocytomas. *Comp Funct Genomics*, **2011**, 245137.
52. Sun, L., Hui, A.M., Su, Q., Vortmeyer, A., Kotliarov, Y., Pastorino, S., Passaniti, A., Menon, J., Walling, J., Bailey, R. *et al.* (2006) Neuronal and glioma-derived stem cell factor induces angiogenesis within the brain. *Cancer Cell*, **9**, 287-300.
53. Lambert, S.R., Witt, H., Hovestadt, V., Zucknick, M., Kool, M., Pearson, D.M., Korshunov, A., Ryzhova, M., Ichimura, K., Jabado, N. *et al.* (2013) Differential expression and methylation of brain developmental genes define location-specific subsets of pilocytic astrocytoma. *Acta Neuropathol*, **126**, 291-301.
54. Griesinger, A.M., Birks, D.K., Donson, A.M., Amani, V., Hoffman, L.M., Waziri, A., Wang, M., Handler, M.H. and Foreman, N.K. (2013) Characterization of distinct immunophenotypes across pediatric brain tumor types. *J Immunol*, **191**, 4880-4888.
55. Runne, H., Kuhn, A., Wild, E.J., Pratyaksha, W., Kristiansen, M., Isaacs, J.D., Regulier, E., Delorenzi, M., Tabrizi, S.J. and Luthi-Carter, R. (2007) Analysis of potential transcriptomic biomarkers for Huntington's disease in peripheral blood. *Proc Natl Acad Sci U S A*, **104**, 14424-14429.
56. Hu, Y., Chopra, V., Chopra, R., Locascio, J.J., Liao, Z., Ding, H., Zheng, B., Matson, W.R., Ferrante, R.J., Rosas, H.D. *et al.* (2011) Transcriptional modulator H2A histone family, member Y (H2AFY) marks Huntington disease activity in man and mouse. *Proc Natl Acad Sci U S A*, **108**, 17141-17146.
57. Consortium, H.D.i. (2012) Induced pluripotent stem cells from patients with Huntington's disease show CAG-repeat-expansion-associated phenotypes. *Cell Stem Cell*, **11**, 264-278.
58. Marchina, E., Misasi, S., Bozzato, A., Ferraboli, S., Agosti, C., Rozzini, L., Borsani, G., Barlati, S. and Padovani, A. (2014) Gene expression profile in fibroblasts of Huntington's disease patients and controls. *J Neurol Sci*, **337**, 42-46.
59. McCourt, A.C., Parker, J., Silajdzic, E., Haider, S., Sethi, H., Tabrizi, S.J., Warner, T.T. and Bjorkqvist, M. (2015) Analysis of White Adipose Tissue Gene Expression Reveals CREB1 Pathway Altered in Huntington's Disease. *J Huntingtons Dis*, **4**, 371-382.
60. Sanchez-Palencia, A., Gomez-Morales, M., Gomez-Capilla, J.A., Pedraza, V., Boyero, L., Rosell, R. and Farez-Vidal, M.E. (2011) Gene expression profiling reveals novel biomarkers in nonsmall cell lung cancer. *Int J Cancer*, **129**, 355-364.
61. Hou, J., Aerts, J., den Hamer, B., van Ijcken, W., den Bakker, M., Riegman, P., van der Leest, C., van der Spek, P., Foekens, J.A., Hoogsteden, H.C. *et al.* (2010) Gene expression-based classification of non-small cell lung carcinomas and survival prediction. *PLoS One*, **5**, e10312.
62. Lu, T.P., Tsai, M.H., Lee, J.M., Hsu, C.P., Chen, P.C., Lin, C.W., Shih, J.Y., Yang, P.C., Hsiao, C.K., Lai, L.C. *et al.* (2010) Identification of a novel biomarker, SEMA5A, for non-small cell lung carcinoma in nonsmoking women. *Cancer Epidemiol Biomarkers Prev*, **19**, 2590-2597.

63. Lu, T.P., Hsiao, C.K., Lai, L.C., Tsai, M.H., Hsu, C.P., Lee, J.M. and Chuang, E.Y. (2015) Identification of regulatory SNPs associated with genetic modifications in lung adenocarcinoma. *BMC Res Notes*, **8**, 92.
64. Zakaria, N., Yusoff, N.M., Zakaria, Z., Lim, M.N., Baharuddin, P.J., Fakiruddin, K.S. and Yahaya, B. (2015) Human non-small cell lung cancer expresses putative cancer stem cell markers and exhibits the transcriptomic profile of multipotent cells. *BMC Cancer*, **15**, 84.
65. Rohrbeck, A., Neukirchen, J., Rosskopf, M., Pardillos, G.G., Geddert, H., Schwalen, A., Gabbert, H.E., von Haeseler, A., Pitschke, G., Schott, M. *et al.* (2008) Gene expression profiling for molecular distinction and characterization of laser captured primary lung cancers. *J Transl Med*, **6**, 69.
66. Badea, L., Herlea, V., Dima, S.O., Dumitrascu, T. and Popescu, I. (2008) Combined gene expression analysis of whole-tissue and microdissected pancreatic ductal adenocarcinoma identifies genes specifically overexpressed in tumor epithelia. *Hepatogastroenterology*, **55**, 2016-2027.
67. Idichi, T., Seki, N., Kurahara, H., Yonemori, K., Osako, Y., Arai, T., Okato, A., Kita, Y., Arigami, T., Mataki, Y. *et al.* (2017) Regulation of actin-binding protein ANLN by antitumor miR-217 inhibits cancer cell aggressiveness in pancreatic ductal adenocarcinoma. *Oncotarget*, **8**, 53180-53193.
68. Pei, H., Li, L., Fridley, B.L., Jenkins, G.D., Kalari, K.R., Lingle, W., Petersen, G., Lou, Z. and Wang, L. (2009) FKBP51 affects cancer cell response to chemotherapy by negatively regulating Akt. *Cancer Cell*, **16**, 259-266.
69. Li, L., Zhang, J.W., Jenkins, G., Xie, F., Carlson, E.E., Fridley, B.L., Bamlet, W.R., Petersen, G.M., McWilliams, R.R. and Wang, L. (2016) Genetic variations associated with gemcitabine treatment outcome in pancreatic cancer. *Pharmacogenet Genomics*, **26**, 527-537.
70. Ellsworth, K.A., Eckloff, B.W., Li, L., Moon, I., Fridley, B.L., Jenkins, G.D., Carlson, E., Brisbin, A., Abo, R., Bamlet, W. *et al.* (2013) Contribution of FKBP5 genetic variation to gemcitabine treatment and survival in pancreatic adenocarcinoma. *PLoS One*, **8**, e70216.
71. Sergeant, G., van Eijsden, R., Roskams, T., Van Duppen, V. and Topal, B. (2012) Pancreatic cancer circulating tumour cells express a cell motility gene signature that predicts survival after surgery. *BMC Cancer*, **12**, 527.
72. Zhang, G., Schetter, A., He, P., Funamizu, N., Gaedcke, J., Ghadimi, B.M., Ried, T., Hassan, R., Yfantis, H.G., Lee, D.H. *et al.* (2012) DPEP1 inhibits tumor cell invasiveness, enhances chemosensitivity and predicts clinical outcome in pancreatic ductal adenocarcinoma. *PLoS One*, **7**, e31507.
73. Zhang, G., He, P., Tan, H., Budhu, A., Gaedcke, J., Ghadimi, B.M., Ried, T., Yfantis, H.G., Lee, D.H., Maitra, A. *et al.* (2013) Integration of metabolomics and transcriptomics revealed a fatty acid network exerting growth inhibitory effects in human pancreatic cancer. *Clin Cancer Res*, **19**, 4983-4993.
74. Donahue, T.R., Tran, L.M., Hill, R., Li, Y., Kovochich, A., Calvopina, J.H., Patel, S.G., Wu, N., Hindoyan, A., Farrell, J.J. *et al.* (2012) Integrative survival-based molecular profiling of human pancreatic cancer. *Clin Cancer Res*, **18**, 1352-1363.

75. Toste, P.A., Li, L., Kadera, B.E., Nguyen, A.H., Tran, L.M., Wu, N., Madnick, D.L., Patel, S.G., Dawson, D.W. and Donahue, T.R. (2015) p85alpha is a microRNA target and affects chemosensitivity in pancreatic cancer. *J Surg Res*, **196**, 285-293.
76. Zheng, B., Liao, Z., Locascio, J.J., Lesniak, K.A., Roderick, S.S., Watt, M.L., Eklund, A.C., Zhang-James, Y., Kim, P.D., Hauser, M.A. *et al.* (2010) PGC-1alpha, a potential therapeutic target for early intervention in Parkinson's disease. *Sci Transl Med*, **2**, 52ra73.
77. Zhang, Y., James, M., Middleton, F.A. and Davis, R.L. (2005) Transcriptional analysis of multiple brain regions in Parkinson's disease supports the involvement of specific protein processing, energy metabolism, and signaling pathways, and suggests novel disease mechanisms. *Am J Med Genet B Neuropsychiatr Genet*, **137B**, 5-16.
78. Lewandowski, N.M., Ju, S., Verbitsky, M., Ross, B., Geddie, M.L., Rockenstein, E., Adame, A., Muhammad, A., Vonsattel, J.P., Ringe, D. *et al.* (2010) Polyamine pathway contributes to the pathogenesis of Parkinson disease. *Proc Natl Acad Sci U S A*, **107**, 16970-16975.
79. Lesnick, T.G., Papapetropoulos, S., Mash, D.C., Ffrench-Mullen, J., Shehadeh, L., de Andrade, M., Henley, J.R., Rocca, W.A., Ahlskog, J.E. and Maraganore, D.M. (2007) A genomic pathway approach to a complex disease: axon guidance and Parkinson disease. *PLoS Genet*, **3**, e98.
80. Wallace, T.A., Prueitt, R.L., Yi, M., Howe, T.M., Gillespie, J.W., Yfantis, H.G., Stephens, R.M., Caporaso, N.E., Loffredo, C.A. and Ambs, S. (2008) Tumor immunobiological differences in prostate cancer between African-American and European-American men. *Cancer Res*, **68**, 927-936.
81. Shan, M., Xia, Q., Yan, D., Zhu, Y., Zhang, X., Zhang, G., Guo, J., Hou, J., Chen, W., Zhu, T. *et al.* (2017) Molecular analyses of prostate tumors for diagnosis of malignancy on fine-needle aspiration biopsies. *Oncotarget*, **8**, 104761-104771.
82. Planche, A., Bacac, M., Provero, P., Fusco, C., Delorenzi, M., Stehle, J.C. and Stamenkovic, I. (2011) Identification of prognostic molecular features in the reactive stroma of human breast and prostate cancer. *PLoS One*, **6**, e18640.
83. Arredouani, M.S., Lu, B., Bhasin, M., Eljanne, M., Yue, W., Mosquera, J.M., Bubley, G.J., Li, V., Rubin, M.A., Libermann, T.A. *et al.* (2009) Identification of the transcription factor single-minded homologue 2 as a potential biomarker and immunotherapy target in prostate cancer. *Clin Cancer Res*, **15**, 5794-5802.
84. He, H., Jazdzewski, K., Li, W., Liyanarachchi, S., Nagy, R., Volinia, S., Calin, G.A., Liu, C.G., Franssila, K., Suster, S. *et al.* (2005) The role of microRNA genes in papillary thyroid carcinoma. *Proc Natl Acad Sci U S A*, **102**, 19075-19080.
85. Rusinek, D., Swierniak, M., Chmielik, E., Kowal, M., Kowalska, M., Cyplinska, R., Czarniecka, A., Piglowski, W., Korfanty, J., Chekan, M. *et al.* (2015) BRAFV600E-Associated Gene Expression Profile: Early Changes in the Transcriptome, Based on a Transgenic Mouse Model of Papillary Thyroid Carcinoma. *PLoS One*, **10**, e0143688.
86. van Tienen, F.H., Praet, S.F., de Feyter, H.M., van den Broek, N.M., Lindsey, P.J., Schoonderwoerd, K.G., de Coo, I.F., Nicolay, K., Prompers, J.J., Smeets, H.J. *et al.* (2012) Physical activity is the key determinant of skeletal muscle mitochondrial function in type 2 diabetes. *J Clin Endocrinol Metab*, **97**, 3261-3269.

87. Patti, M.E., Butte, A.J., Crunkhorn, S., Cusi, K., Berria, R., Kashyap, S., Miyazaki, Y., Kohane, I., Costello, M., Saccone, R. *et al.* (2003) Coordinated reduction of genes of oxidative metabolism in humans with insulin resistance and diabetes: Potential role of PGC1 and NRF1. *Proc Natl Acad Sci U S A*, **100**, 8466-8471.
88. Greco, S., Fasanaro, P., Castelvecchio, S., D'Alessandra, Y., Arcelli, D., Di Donato, M., Malavazos, A., Capogrossi, M.C., Menicanti, L. and Martelli, F. (2012) MicroRNA dysregulation in diabetic ischemic heart failure patients. *Diabetes*, **61**, 1633-1641.
89. Taneera, J., Lang, S., Sharma, A., Fadista, J., Zhou, Y., Ahlqvist, E., Jonsson, A., Lyssenko, V., Vikman, P., Hansson, O. *et al.* (2012) A systems genetics approach identifies genes and pathways for type 2 diabetes in human islets. *Cell Metab*, **16**, 122-134.
90. Taneera, J., Fadista, J., Ahlqvist, E., Zhang, M., Wierup, N., Renstrom, E. and Groop, L. (2013) Expression profiling of cell cycle genes in human pancreatic islets with and without type 2 diabetes. *Mol Cell Endocrinol*, **375**, 35-42.
91. Kanatsuna, N., Taneera, J., Vaziri-Sani, F., Wierup, N., Larsson, H.E., Delli, A., Skarstrand, H., Balhuizen, A., Bennet, H., Steiner, D.F. *et al.* (2013) Autoimmunity against INS-IGF2 protein expressed in human pancreatic islets. *J Biol Chem*, **288**, 29013-29023.
92. Hanzelmann, S., Wang, J., Guney, E., Tang, Y., Zhang, E., Axelsson, A.S., Nenonen, H., Salehi, A.S., Wollheim, C.B., Zetterberg, E. *et al.* (2015) Thrombin stimulates insulin secretion via protease-activated receptor-3. *Islets*, **7**, e1118195.
93. Knebel, B., Kotzka, J., Lehr, S., Hartwig, S., Avci, H., Jacob, S., Nitzgen, U., Schiller, M., Marz, W., Hoffmann, M.M. *et al.* (2013) A mutation in the c-fos gene associated with congenital generalized lipodystrophy. *Orphanet J Rare Dis*, **8**, 119.
94. Nguyen, T.M., Shafi, A., Nguyen, T. and Draghici, S. (2019) Identifying significantly impacted pathways: a comprehensive review and assessment. *Genome Biol*, **20**, 203.
95. Desai, N., Neyaz, A., Szabolcs, A., Shih, A.R., Chen, J.H., Thapar, V., Nieman, L.T., Solovyov, A., Mehta, A., Lieb, D.J. *et al.* (2020) Temporal and spatial heterogeneity of host response to SARS-CoV-2 pulmonary infection. *Nat Commun*, **11**, 6319.
96. Overmyer, K.A., Shishkova, E., Miller, I.J., Balnis, J., Bernstein, M.N., Peters-Clarke, T.M., Meyer, J.G., Quan, Q., Muehlbauer, L.K., Trujillo, E.A. *et al.* (2021) Large-Scale Multi-omic Analysis of COVID-19 Severity. *Cell Syst*, **12**, 23-40 e27.
97. Blanco-Melo, D., Nilsson-Payant, B.E., Liu, W.C., Uhl, S., Hoagland, D., Moller, R., Jordan, T.X., Oishi, K., Panis, M., Sachs, D. *et al.* (2020) Imbalanced Host Response to SARS-CoV-2 Drives Development of COVID-19. *Cell*, **181**, 1036-1045 e1039.
98. Daamen, A.R., Bachali, P., Owen, K.A., Kingsmore, K.M., Hubbard, E.L., Labonte, A.C., Robl, R., Shrotri, S., Grammer, A.C. and Lipsky, P.E. (2021) Comprehensive transcriptomic analysis of COVID-19 blood, lung, and airway. *Sci Rep*, **11**, 7052.
99. Mulay, A., Konda, B., Garcia, G., Jr., Yao, C., Beil, S., Villalba, J.M., Koziol, C., Sen, C., Purkayastha, A., Kolls, J.K. *et al.* (2021) SARS-CoV-2 infection of primary human lung epithelium for COVID-19 modeling and drug discovery. *Cell Rep*, **35**, 109055.
100. Alexeyenko, A., Lee, W., Pernemalm, M., Guegan, J., Dessen, P., Lazar, V., Lehtio, J. and Pawitan, Y. (2012) Network enrichment analysis: extension of gene-set enrichment analysis to gene networks. *BMC Bioinformatics*, **13**, 226.

101. Jeggari, A., Alekseenko, Z., Petrov, I., Dias, J.M., Ericson, J. and Alexeyenko, A. (2018) EviNet: a web platform for network enrichment analysis with flexible definition of gene sets. *Nucleic Acids Res*, **46**, W163-W170.
102. Jeggari, A. and Alexeyenko, A. (2017) NEArender: an R package for functional interpretation of 'omics' data via network enrichment analysis. *BMC Bioinformatics*, **18**, 118.
103. Draghici, S., Khatri, P., Bhavsar, P., Shah, A., Krawetz, S.A. and Tainsky, M.A. (2003) Onto-Tools, the toolkit of the modern biologist: Onto-Express, Onto-Compare, Onto-Design and Onto-Translate. *Nucleic Acids Res*, **31**, 3775-3781.
104. Voichita, C., Donato, M. and Draghici, S. (2012) Incorporating Gene Significance in the Impact Analysis of Signaling Pathways. *2012 11th International Conference on Machine Learning and Applications*, **1**, 126-131.
105. Nguyen, H., Tran, D., Galazka, J.M., Costes, S.V., Beheshti, A., Petereit, J., Draghici, S. and Nguyen, T. (2021) CPA: a web-based platform for consensus pathway analysis and interactive visualization. *Nucleic Acids Res*, **49**, W114-W124.
106. Ahsan, S. and Draghici, S. (2017) Identifying Significantly Impacted Pathways and Putative Mechanisms with iPathwayGuide. *Curr Protoc Bioinformatics*, **57**, 7 15 11-17 15 30.
107. Tarca, A.L., Draghici, S., Khatri, P., Hassan, S.S., Mittal, P., Kim, J.S., Kim, C.J., Kusanovic, J.P. and Romero, R. (2009) A novel signaling pathway impact analysis. *Bioinformatics*, **25**, 75-82.
108. Sales, G., Calura, E., Cavalieri, D. and Romualdi, C. (2012) graphite - a Bioconductor package to convert pathway topology to gene network. *BMC Bioinformatics*, **13**, 20.
109. Gu, Z., Liu, J., Cao, K., Zhang, J. and Wang, J. (2012) Centrality-based pathway enrichment: a systematic approach for finding significant pathways dominated by key genes. *BMC Syst Biol*, **6**, 56.
110. Gu, Z. and Wang, J. (2013) CePa: an R package for finding significant pathways weighted by multiple network centralities. *Bioinformatics*, **29**, 658-660.
111. Tarca, A.L., Draghici, S., Bhatti, G. and Romero, R. (2012) Down-weighting overlapping genes improves gene set analysis. *BMC Bioinformatics*, **13**, 136.
112. Efron, B. and Tibshirani, R. (2007) On testing the significance of sets of genes. *The Annals of Applied Statistics*, **1**, 107-129, 123.
113. Subramanian, A., Tamayo, P., Mootha, V.K., Mukherjee, S., Ebert, B.L., Gillette, M.A., Paulovich, A., Pomeroy, S.L., Golub, T.R., Lander, E.S. *et al.* (2005) Gene set enrichment analysis: a knowledge-based approach for interpreting genome-wide expression profiles. *Proc Natl Acad Sci U S A*, **102**, 15545-15550.
114. Liao, Y., Wang, J., Jaehnig, E.J., Shi, Z. and Zhang, B. (2019) WebGestalt 2019: gene set analysis toolkit with revamped UIs and APIs. *Nucleic Acids Res*, **47**, W199-W205.
115. Wu, T., Hu, E., Xu, S., Chen, M., Guo, P., Dai, Z., Feng, T., Zhou, L., Tang, W., Zhan, L. *et al.* (2021) clusterProfiler 4.0: A universal enrichment tool for interpreting omics data. *Innovation (Camb)*, **2**, 100141.
116. Khatri, P., Draghici, S., Ostermeier, G.C. and Krawetz, S.A. (2002) Profiling gene expression using onto-express. *Genomics*, **79**, 266-270.

117. Draghici, S., Khatri, P., Martins, R.P., Ostermeier, G.C. and Krawetz, S.A. (2003) Global functional profiling of gene expression. *Genomics*, **81**, 98-104.
118. Al-Shahrour, F., Diaz-Uriarte, R. and Dopazo, J. (2004) FatiGO: a web tool for finding significant associations of Gene Ontology terms with groups of genes. *Bioinformatics*, **20**, 578-580.
119. Beissbarth, T. and Speed, T.P. (2004) GOstat: find statistically overrepresented Gene Ontologies within a group of genes. *Bioinformatics*, **20**, 1464-1465.
120. Sherman, B.T., Hao, M., Qiu, J., Jiao, X., Baseler, M.W., Lane, H.C., Imamichi, T. and Chang, W. (2022) DAVID: a web server for functional enrichment analysis and functional annotation of gene lists (2021 update). *Nucleic Acids Res*, **50**, W216-W221.
121. Huang da, W., Sherman, B.T. and Lempicki, R.A. (2009) Systematic and integrative analysis of large gene lists using DAVID bioinformatics resources. *Nat Protoc*, **4**, 44-57.
